# Immune and ionic mechanisms mediating the effect of dexamethasone in severe COVID-19

**DOI:** 10.3389/fimmu.2023.1143350

**Published:** 2023-03-24

**Authors:** Ameet A. Chimote, Abdulaziz O. Alshwimi, Martina Chirra, Vaibhavkumar S. Gawali, Margaret V. Powers-Fletcher, Kristin M. Hudock, Laura Conforti

**Affiliations:** ^1^Department of Internal Medicine, Division of Nephrology, University of Cincinnati, Cincinnati, OH, United States; ^2^Department of Internal Medicine, Division of Infectious Diseases, University of Cincinnati, Cincinnati, OH, United States; ^3^Department of Internal Medicine, Division of Pulmonary, Critical Care and Sleep Medicine, University of Cincinnati, Cincinnati, OH, United States; ^4^Department of Pediatrics, Division of Pulmonary Biology, Cincinnati Children’s Hospital Medical Center, Cincinnati, OH, United States

**Keywords:** COVID-19, ion channels, interferon signaling, severe COVID-19, cytokine storm, dexamethasone, severe COVID-19 immune signaling

## Abstract

**Introduction:**

Severe COVID-19 is characterized by cytokine storm, an excessive production of proinflammatory cytokines that contributes to acute lung damage and death. Dexamethasone is routinely used to treat severe COVID-19 and has been shown to reduce patient mortality. However, the mechanisms underlying the beneficial effects of dexamethasone are poorly understood.

**Methods:**

We conducted transcriptomic analysis of peripheral blood mononuclear cells (PBMCs) from COVID-19 patients with mild disease, and patients with severe COVID-19 with and without dexamethasone treatment. We then treated healthy donor PBMCs in vitro with dexamethasone and investigated the effects of dexamethasone treatment ion channel abundance (by RT-qPCR and flow cytometry) and function (by electrophysiology, Ca2+ influx measurements and cytokine release) in T cells.

**Results:**

We observed that dexamethasone treatment in severe COVID-19 inhibited pro-inflammatory and immune exhaustion pathways, circulating cytotoxic and Th1 cells, interferon (IFN) signaling, genes involved in cytokine storm, and Ca^2+^ signaling. Ca^2+^ influx is regulated by Kv1.3 potassium channels, but their role in COVID-19 pathogenesis remains elusive. Kv1.3 mRNA was increased in PBMCs of severe COVID-19 patients, and was significantly reduced in the dexamethasone-treated group. In agreement with these findings, in vitro treatment of healthy donor PBMCs with dexamethasone reduced Kv1.3 abundance in T cells and CD56dimNK cells. Furthermore, functional studies showed that dexamethasone treatment significantly reduced Kv1.3 activity, Ca2+ influx and IFN-g production in T cells.

**Conclusion:**

Our findings suggest that dexamethasone attenuates inflammatory cytokine release via Kv1.3 suppression, and this mechanism contributes to dexamethasone-mediated immunosuppression in severe COVID-19.

## 1 Introduction

Coronavirus disease 2019 (COVID-19), caused by severe acute respiratory syndrome coronavirus 2 (SARS-CoV-2), is a public health emergency that has affected millions globally. Since the outset of the COVID-19 pandemic, there has been a concerted global research effort to understand the nature of the disease caused by SARS-CoV-2, decipher the complex immune responses to SARS-CoV-2 infection, and develop effective therapeutics and vaccines (1–3). Inflammatory cytokines, produced and released by activated innate and adaptive immune cells, are essential for anti-viral immune responses, including the response to SARS-CoV-2. However, in severe respiratory COVID-19, infection of lung tissue by SARS-CoV-2 triggers the cross-talk between epithelial cells and innate and adaptive immune cells leading to pathologically uncontrolled levels of proinflammatory cytokines, i.e. “cytokine storm”. The cytokine storm ultimately results in respiratory failure and unfavorable patient outcomes (4, 5). Indeed, severe COVID-19 is characterized by a marked increase in the production of proinflammatory cytokines such as interleukin-6 (IL-6), interferon-γ (IFN-γ), and IL-17, diminished antiviral type I and type III interferon (IFN) responses, increased immune cell exhaustion and decreased cytotoxicity (3, 6, 7). Understanding the molecular mechanisms responsible for these aberrant immune responses in severe COVID-19 patients is critical to the development of new therapeutics that may prevent cytokine storm and mortality.

Dexamethasone, a corticosteroid drug with exclusively glucocorticoid activity, is currently administered as standard of care for severe COVID-19. Results from the RECOVERY (Randomized Evaluation of COVID-19 Therapy) clinical trial in mid-2020 showed that dexamethasone reduces mortality by one-third in severely ill COVID-19 patients requiring mechanical ventilation (8). Dexamethasone is an anti-inflammatory drug commonly prescribed to treat several diseases including autoimmune and inflammatory disorders, cancers and acute respiratory distress syndrome (ARDS) (9–12). In severely ill COVID-19 patients, dexamethasone is purported to reduce cytokine storms by inhibiting immune cells like T, B, Natural Killer (NK) and dendritic cells and specifically by suppressing their abundance and function, including T cell receptor signaling and cytokine release (12–14). However, there remains a critical gap, in understanding the molecular mechanisms underlying the effects of dexamethasone that lead to attenuation of COVID-19 severity.

Immune cell function, including cytokine production and release is controlled by Ca^2+^ signaling that is regulated by the concerted function of ion channels, transporters, and pumps. Activation-induced Ca^2+^ influx in immune cells occurs through Ca^2+^ release–activated Ca^2+^ (CRAC) channels that consist of two subunits: a plasma membrane localized pore-forming subunit, Orai1, and an endoplasmic reticulum-localized Ca^2+^ sensor, the stromal interaction molecule 1 (Stim1) (15, 16). Ca^2+^ influx through CRAC channels is facilitated by the voltage-gated Kv1.3 and the Ca^2+^ dependent KCa3.1 potassium (K^+^) channels, which control the membrane potential and hence the driving force for Ca^2+^ influx into the cell (15, 17). Increased intracellular Ca^2+^ is necessary for activation of transcription factors that control the expression of molecules involved in multiple immune effector functions, including cytokine production and release, cytotoxicity and proliferation (15). Ca^2+^ signaling is required for expression of the majority of T cell activation genes (15, 17). Previously published studies show that blockade of ion channel function in T cells resulted in decreased cytotoxicity, motility, and cytokine release (18–22). Although an effective adaptive immune response mediated by cytotoxic CD8^+^ T cells against SARS-CoV-2 is critical to controlling viral dissemination and lung damage, the contribution of the ion channels, including Kv1.3, in regulating the effector functions of T cells and other immune cells involved in the response against SARS-CoV-2 is yet to be investigated. Interestingly, dexamethasone has been shown to downregulate Kv1.3 channels in a human macrophage cell line and in Jurkat T cells (13, 23). Therefore, it would be important to investigate if ion channels, especially Kv1.3 play an important role in mediating the therapeutic effect of dexamethasone treatment in severe COVID-19.

In this study, we performed a comparative transcriptomic analysis in peripheral blood mononuclear cells (PBMCs) from patients with mild and severe COVID-19 disease with and without dexamethasone treatment. We observed that not only severe illness altered key immune signaling pathways involved in inflammation, cytokine release, exhaustion, and antiviral immunity in PBMCs but also that these alterations were associated with an increase in Kv1.3 channel expression. Moreover, by demonstrating the differentially regulated pathways as a result of dexamethasone treatment in severe COVID-19 patients, we provide evidence that decreased expression of Kv1.3 channels in PBMCs mediates the immunosuppressive effect of dexamethasone.

## 2 Materials and methods

### 2.1 Study design

This study was designed to investigate the immune and molecular mechanisms involved in COVID-19 disease progression and determine the beneficial effects of dexamethasone in severely ill patients. This study was conducted in PBMCs from patients with mild and severe COVID-19. For the latter group, we utilized samples from patients that either were treated with dexamethasone or not. We used cryopreserved PBMCs from unexposed healthy donors (collected before the arrival of SARs-Cov-2 in the United States) as controls. We conducted transcriptomic experiments and identified the antiviral immune response pathways and genes that were differentially expressed between the following groups: 1) mild COVID-19 patients vs. healthy individuals, 2) severe vs. mild COVID-19 patients, and 3) severe COVID-19 patients with or without dexamethasone treatment. We then conducted a correlation analysis utilizing our gene sets. This analysis identified Ca^2+^ fluxes-related functions among the biological functions that were modified by dexamethasone-treatment in patients with severe COVID-19. Since Ca^2+^ fluxes in immune cells are controlled by ion channels, we performed RT-qPCR experiments to quantify the ion channel expression in our patient cohorts. We confirmed the inhibitory effect of dexamethasone on ion channel abundance (by RT-qPCR and flow cytometry) by conducting *in vitro* experiments with healthy donor PBMCs. Next, we determined the functional significance of Kv1.3 alterations due to dexamethasone in CD8^+^ T cells by measuring the channel activity (by electrophysiology) and IFN-γ production. The criteria for patient inclusion in our study are described in detail below. The samples were de-identified, and the sample size was sufficient to detect statistical significance between the experimental groups. None of the samples were excluded from analysis, and investigators were not blinded during experiments. Statistical methods used in this study are summarized in the “Statistical analysis” section, and the P values and statistical methods used for individual experiments are provided in the individual figures and figure legends.

### 2.2 Patient samples (human samples and subjects)

De-identified PBMCs samples from patients with 1) mild COVID-19, 2) severe COVID-19, and 3) severe COVID-19 treated with dexamethasone were obtained from the Cincinnati COVID-19 Biorepository. Adult subjects who presented to the University of Cincinnati Medical Center (UCMC) with at least one symptom of COVID-19 (shortness of breath, fever, cough) and/or a positive RT-PCR test for SARS-CoV-2 provided written informed consent to participate in this study. Subjects with mild disease were outpatients or were seen in the ER only and were never admitted at UCMC for COVID-19. Individuals who were defined as having severe disease were patients hospitalized in the UCMC (requiring admission to the ICU or Medical Step-down units) with signs of respiratory distress and requiring O_2_ within 7 days of their acute presentation. The individuals included in the severe COVID-19 treated with dexamethasone cohort were severe cases (defined above) who were treated with 6 mg of dexamethasone per day between 1-7 days of a positive COVID-19 diagnosis. Exclusion criteria for all groups included a diagnosis of cancer or autoimmune disease, and for mild and severe groups (dexamethasone-untreated), we also had an additional exclusion criterion that the subjects should not have received dexamethasone or any other corticosteroid. Patients included in our study were in the age range of 38-75 years and were included regardless of race, ethnicity, gender, pregnancy status, or any pre-existing conditions (except cancer or autoimmunity as mentioned earlier). The samples for the mild and severe COVID-19 patient cohorts were collected between early May and early July 2020, when dexamethasone treatment for severe cases was not the prevalent standard of care for severe illness, prior to the published findings of the RECOVERY trial (8). Only one individual in the severe COVID-19 patient cohort received Remdesivir, as widespread access to this drug was limited early on during the pandemic. The samples for severe COVID-19 patients that received dexamethasone were collected between mid-August and mid-September 2020. All of the demographic, clinical, laboratory and treatment data from the date closest to the sample collection were extracted from the patients’ electronic medical records and compiled in a centralized database created by the Center for Health Informatics at the University of Cincinnati and are presented in **Tables S1**, **S2**. In **Table S2**, all of the severe COVID-19 patients included in the dexamethasone-treated cohort received at least 6 mg of dexamethasone for 5-6 days before the samples were collected. Interestingly, mortality was not reported in any of the individuals included in this study.

Whole blood was freshly collected from all subjects and immediately processed. PBMCs were isolated from the whole blood by Ficoll-Paque density gradient centrifugation as described previously and cryopreserved at the University of Cincinnati Biorepository until further use (21).

As healthy controls for this study, we used cryopreserved PBMCs that were isolated from discarded blood units from Hoxworth Blood Center (University of Cincinnati) for prior unrelated studies in our laboratory between late 2016 and mid-2019. We considered these donors as unexposed healthy controls as the first cases of SARS-CoV-2 were documented in the state of Ohio on March 9, 2020. Demographic information about these samples is not available.

Informed consent was obtained from all COVID-19 patients and healthy donors participating in the study. Sample collection for the Cincinnati COVID-19 Biorepository and informed patient consent for the study were approved under IRB # 2020-0329, while sample collection from blood obtained from the Hoxworth Blood Center was approved under IRB # 2013-2516 by the University of Cincinnati Institutional Review Board.

### 2.3 Sample processing

The cryopreserved PBMCs from COVID-19 patient cohorts and unexposed healthy controls were thawed in a 37°C water bath, centrifuged at 300 X g for 10 minutes at room temperature, and the cell pellets were resuspended in RPMI 1640 medium supplemented with 10% human serum, penicillin (200 U/ml), streptomycin (200 μg/ml), 1 mM L-glutamine, and 10 mM Hepes (all from ThermoFisher) and maintained overnight (~12 hours) in a cell culture incubator at 37°C supplemented with 5% CO2. The viability of the samples (**Table S3**) was assessed by flow cytometry using Zombie UV Live/Dead stain (Biolegend) prior to RNA isolation for NanoString and RT-qPCR assays.

### 2.4 Immune cell profiling, immune pathway, and gene expression analysis with NanoString nCounter

Transcriptomic analysis on COVID-19 patients (mild, severe +/- dexamethasone) and unexposed healthy donor PBMCs was performed using NanoString nCounter platform (NanoString Technologies). Total RNA was isolated from the samples using the E.Z.N.A. total RNA isolation Kit (Omega Bio-tek) and quantified using a NanoDrop 2000 spectrophotometer (ThermoFisher Scientific). The RNA quality was analyzed using an Agilent 2100 Bioanalyzer (Agilent); and concentrated using the RNA Clean and Concentrator kit (Zymo Corp.). All of our processed RNA samples fulfilled the following qualitative criteria to be eligible for NanoString analysis: 260:280 ratio > 1.8, 260:230 ratio > 2.0, and DV-200 (percentage of nucleotide fragments >200 bp) > 30% (22). We analyzed 100 ng of RNA from four mild, three severe, and four severe COVID-19 patients treated with dexamethasone, along with five unexposed healthy donors using the human Host Response CodeSet panel (NanoString), which includes 773 genes covering the host immune response to infectious diseases, and 12 internal reference genes for data normalization. The RNA samples were hybridized as per the manufacturer’s instructions, and each sample was read in a multiplexed reaction with the reference (positive, negative controls, and housekeeping genes) probes on the NanoString nCounter Flex instrument.

### 2.5 NanoString data analysis

The raw gene expression data (counts) were calculated through the Basic Analysis of the nSolver software (version 4.0, NanoString) and then imported into the nSolver Advanced Analysis plug-in (version 2.0) for cell type profiling, pathway score analysis, and differential gene expression; and Rosalind software (OnRamp Bio) for normalized gene expression data. The counts were normalized to the geometric means of internal housekeeping gene probes. Principal components analysis was performed on the normalized counts of the individual genes using GraphPad Prism (version 9.1.2). Relative cell type abundance was calculated using the nSolver Advanced Analysis software as described by us earlier using the method described by Danaher et al., and data are presented as “cell scores” (22, 24). Pathway scores were determined using the nSolver advanced analysis, which condensed each sample’s gene expression profile into a small set of pathway scores, which were derived using the first principal component of each gene set’s data and are oriented such that a higher pathway score corresponds to mostly higher abundance of the constituent genes that define each individual pathway. To determine changes in the pathway scores in the patient cohorts, we performed pairwise student’s t-tests between 1) healthy controls and mild COVID-19, 2) mild and severe COVID-19, and 3) severe COVID-19 +/- dexamethasone. We identified the pathways that were significantly changed by dexamethasone in severe COVID-19 and determined the changes in the abundance of the individual genes that constituted these pathways by performing t-tests on the normalized counts of the individual genes between 1) mild and severe COVID-19 and 2) severe COVID-19 +/- dexamethasone. Heatmaps were generated for cell scores, pathway scores, and normalized counts using Morpheus open-source software (https://software.broadinstitute.org/morpheus). Differential gene expression with dexamethasone treatment in severe COVID-19 was performed by pairwise comparison between severely ill patients +/- dexamethasone using the nSolver advanced analysis. P value was adjusted in the nSolver advanced analysis using the Benjamini-Yekutieli method with the false discovery threshold set at 0.05, and the data were plotted as a volcano plot by graphing each target’s -log_10_ (p-value) and log_2_ (fold change) using GraphPad Prism. The normalized gene count data was further used to perform network visualization to determine whether dexamethasone treatment in severe COVID-19 patients altered any biological processes associated with Ca^2+^ signaling. For this analysis, we first calculated the differential gene expression for each gene in the NanoString panel using the open-source software AltAnalyze (http://www.AltAnalyze.org). We defined the genes with a P value < 0.05 and a fold change > 1.5 (or < 1.5) as significantly differentially expressed. We identified the genes increased (> 1.5 fold change) in severe COVID-19 as compared to mild disease and genes decreased (< 1.5 fold change) by dexamethasone treatment in severe illness. Further functional enrichment analysis was performed on these differentially expressed genes using g: Profiler (https://biit.cs.ut.ee/gprofiler) by evaluating them for enrichment of Gene Ontology (GO) term- Biological Processes (25). Visual interaction networks for enriched Biological Processes were generated using Cytoscape (version 3.9.1) (26). We generated visual interaction networks for all of the calcium ion signaling-related pathways decreased by dexamethasone treatment in severe disease.

### 2.6 Healthy donor cell isolation, *in vitro* dexamethasone treatment and CD8^+^ T cell activation

For experiments involving *in vitro* dexamethasone treatment, we obtained discarded blood units from Hoxworth Blood Center. Demographic information about these samples is not available. PBMCs were isolated from whole blood as described earlier by Ficoll-Paque density gradient centrifugation (GE Healthcare Bio-Sciences) (21). CD8^+^ T cells were isolated from PBMCs by negative selection using the EasySep Human CD8^+^ T Cell Enrichment Kit (STEMCELL Technologies Inc.) as per the manufacturer’s protocol. The PBMCs and CD8^+^ T cells were maintained in RPMI 1640 medium supplemented with 10% human serum, penicillin (200 U/ml), streptomycin (200 μg/ml), 1 mM L-glutamine, and 10 mM Hepes and treated with dexamethasone (Millipore Sigma). Stock solution (1 mM) of dexamethasone was prepared in absolute ethanol (Fisher Scientific) and added to the cultured PBMCs and CD8^+^ T cells (~3 X 10^6^ cells) for 24 and 48 h, at final working concentrations of 1 μM and 0.1 μM. Viability of cells post-dexamethasone treatment was determined by trypan blue exclusion (**Figure S1**). Some of the treated PBMCs (~ 1 X 10^6^ cells) were cryopreserved (CryoStor CS10 cryopreservation medium, STEMCELL Technologies) and stored in liquid nitrogen until used for flow cytometry experiments. The remaining PBMCs and all of the CD8^+^ T cells were used immediately for RT-qPCR and electrophysiology experiments. For the experiments involving Ca^2+^ and IFN-γ measurements, CD8^+^ T cells were activated with plate-bound mouse anti-human CD3 (10 μg/ml) and mouse anti-human CD28 (10 μg/ml) antibodies (BioLegend) as previously described (18, 22).

### 2.7 RT-qPCR

Total RNA was isolated from PBMCs using the E.Z.N.A. total RNA isolation Kit (Omega Bio-tek). 300 ng of RNA was used to synthesize complementary DNA (cDNA) using the LunaScript^®^ RT SuperMix Kit (New England Biolabs) as per the manufacturer’s instructions. Predesigned primers for RT-qPCR were obtained using TaqMan Gene Expression Assays (Applied Biosystems, ThermoFisher) to detect the expression of *KCNA3* (assay ID: Hs00704943_s1), *KCNN4* (assay ID: Hs01069779_m1), *Orai1* (assay ID: Hs03046013_m1), *Stim1* (assay ID: Hs00963373_m1) and *18S rRNA* (assay ID: Hs99999901_s1). The RT-qPCR reactions were set up in a 96-well plate by adding 30 ng of cDNA, 1× TaqMan Gene Expression Master Mix (Applied Biosystems, ThermoFisher), and 1 μl of TaqMan Gene Expression Assay primers. All samples were run in triplicates. *18S rRNA* was used as an internal control. RT-qPCR was cycled in QuantStudio Real-Time PCR Systems (Applied Biosystems, ThermoFisher). C_T_ values were measured using QuantStudio Design & Analysis software v 1.5.1 (Applied Biosystems). C_T_ values for *KCNA3*, *KCNN*4, *Orai*, and *Stim1* were normalized against measured C_T_ values for *18S rRNA*, and the ΔΔC_T_ values were calculated as described previously (20). Relative quantity (RQ) values, which represent the fold change in the gene expression, were calculated as the 2^−ΔΔC^_T_ values as compared to control (healthy donor PBMCs) samples.

### 2.8 Flow cytometry

For phenotyping, cryopreserved PBMCs (~1x10^6^ per condition) from healthy donors +/- dexamethasone were thawed, resuspended in 100 μl PBS and stained with Zombie UV Live/Dead stain for 20 min. Cells were washed with cell staining buffer (Biolegend) and fixed with 4% paraformaldehyde for 30 min. Cells were stained for surface guinea pig anti- Kv1.3 (Alomone Labs) and Orai1 (ATTO-633, Alomone Labs) primary antibodies overnight at 4°C in the dark. The next day, cells were washed twice in cell staining buffer, followed by a secondary anti-guinea pig (Alexa Fluor 555 goat anti-guinea pig IgG; ThermoFisher) antibody. Cells were then stained with an antibody cocktail for surface stains (CD3, CD4, CD8, CD19, CD56, CD16, and CD14 antibodies) and acquired on a flow cytometer. A complete list of antibodies used in the phenotyping staining panel are presented in **Table S4**. We have previously confirmed the specificity of the Kv1.3 and Orai1 antibodies used this this study (19, 27). All samples were acquired on a BD Fortessa flow cytometer (BD Biosciences) and analyzed with FlowJo software (BD Biosciences). Unstained and single-stained PBMCs from healthy donors, along with UltraComp eBeads (ThermoFisher), were used for compensation.

### 2.9 Electrophysiology

Kv1.3 currents were recorded from CD8^+^ T cells in whole-cell voltage clamp configuration using an AxoPatch 200B Amplifier (Molecular devices). Pipettes were formed from Borosilicate glass (TW150F-4, World Precision Instruments) with a P-97 horizontal puller (Sutter Instruments) and had a resistance between 4 and 7 MΩ when filled with intracellular pipette solution containing (in mmol/L) 140 KF, 55 EGTA, 5 CaCl_2_, 10 MgCl_2_, and 10 HEPES [pH 7.22; (19)]. The external solution contained (in mmol/L) 145 NaCl, 5 KCl, 1 MgCl_2_, 2.5 CaCl_2_, 5.5 glucose, and 10 HEPES (pH 7.4,all reagents from Millipore-Sigma). Kv1.3 currents were elicited by 800-ms step pulse depolarization from -80mV to +50 mV. Data were acquired using pCLAMP 8.0 software (Molecular Devices) through a 16-bit A-D/D-A interface (Digidata1320A, Molecular Devices). Data were low pass filtered frequency at 2 kHz and digitalized at 100 kHz. The amplitude of the peak current was determined at +50 mV. The verity of Kv1.3 currents was confirmed by blocking the channels by ShK-Dap22 (Bachem), a specific Kv1.3 channel blocker (28).

### 2.10 Calcium measurements

Intracellular Ca^2+^ was measured using the Ca^2+^ add-back method (19, 29). Briefly, 0.1-1 X 10^6^ freshly isolated CD8^+^ T cells treated with either dexamethasone or ShK-Dap22 (vehicle- treated cells used as controls) and activated as described in section 2.6, were loaded with 1:1000 fold of 2 mg/ml Indo-1 AM ratiometric dye (ThermoFisher) and 0.015% Pluronic F-127 (ThermoFisher) in Hank’s balanced salt solution containing (in mM) 1 CaCl_2_, 1 MgCl_2_ and 1% FBS for 30 min at 37°C, then washed three times in Hank’s balanced salt solution supplemented with 10 mM HEPES (pH 7.0) and 1% FBS. Prior to measurements, cells were resuspended in a Ca^2+^-depleted solution prepared from the HBSS/HEPES solution mentioned above and supplemented with 0.5 mM EGTA (pH 7.4). Samples were kept in a 37°C water bath until analysis. The Indo-1 fluorescence ratio (indicative of the intracellular Ca^2+^ levels) in CD8^+^ T cells was measured by flow cytometry on a BD Fortessa Flow cytometer (BD Biosciences), using instrument set up acquisition parameters and experimental protocol as described by us earlier (29). The following protocol was used to measure changes in intracellular Ca^2+^: cells were first exposed to thapsigargin (TG, 1 μM) in 0 mM Ca^2+^ solution followed by 2 mM Ca^2+^-containing solution. Analysis of the kinetics was performed using FlowJo software (BD Biosciences). Ca^2+^ fold change was measured as the ratio between the peak intensity ratio of Indo-1 upon addition of 2 mM Ca^2+^ and the mean baseline Indo-1 ratio at 0 mM Ca^2+^ (prior to TG addition). We also calculated the area under the curve (AUC), an estimate for the average Ca^2+^ influx into the cell, for the part of Ca^2+^-response curve after addition of 2 mM Ca^2+^ solution.

### 2.11 IFN-γ measurements

For measuring IFN-γ release, cryopreserved PBMCs from severely ill COVID-19 patients as well as from healthy donors were thawed and rested overnight in the presence of 10 ng/ml IL-2 (Biolegend). The following day, viability was determined by trypan blue exclusion, and CD8^+^ T cells were isolated as described in section 2.6. The concentration of the CD8^+^ T cells was adjusted to 1 X 10^6^ cells/ml and cells were then treated with 0.1 and 1 mM dexamethasone, 10 nM and 100 nM ShK-Dap22, while cells treated with vehicle served as controls, and were activated for 48 h with plate-bound anti-human CD3 and anti-human CD28 antibodies as described in section 2.6. After 48 h, IFN-γ levels in cell culture supernatants were detected by using enzyme-linked immunosorbent assay (ELISA) (Human IFN gamma uncoated ELISA kit, ThermoFisher) according to the manufacturer’s instructions.

### 2.12 Statistical analysis

Statistical analyses were performed using either Student’s t test (paired or unpaired) and analysis of variance (ANOVA). The normality of sample distribution was assessed by Shapiro-Wilk test, and where the samples failed normality, comparisons were performed by Mann-Whitney rank sum test or ANOVA on ranks. *Post hoc* testing on ANOVA was performed by multiple pairwise comparison procedures using either Holm-Sidak, Tukey, or Dunn’s methods. Statistical analysis was performed using SigmaPlot 13.0 (Systat Software Inc.) and GraphPad Prism 9.0 (GraphPad Software LLC.). P ≤ 0.05 was defined as statistically significant. The statistical tests applied to detect significance for our NanoString experiments are described in the NanoString data analysis section. For NanoString data analysis, due to the small sample size and low power for statistical analysis, we did not perform multiple comparison adjustments and have reported the non-adjusted P-values in the relevant data figures and tables. The appropriate statistical tests, along with their significance values for all of the data, are described in the individual figure legends.

## 3 Results

### 3.1 Immune profiles of COVID-19 associated with disease progression and dexamethasone treatment

**Figure 1:**
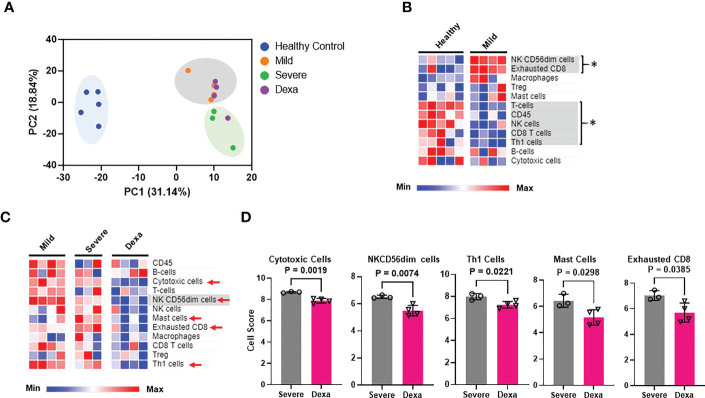
Immune cell profiling of PBMCs from healthy controls, mild and severe COVID-19 patients and severe COVID-19 patients treated with dexamethasone. **(A)** Principal components analysis (PCA) of NanoString transcriptomic data. **(B)** Heatmap depicting immune cell abundance in PBMCs from healthy donors (n=5) and mild COVID-19 patients (n=4). Significantly altered cell types are highlighted in gray and represented by *. **(C)** Heatmap depicting abundance of immune cell types in PBMCs from patients with mild (n=4), severe COVID-19 (Severe, n=3), and severe COVID-19 + dexamethasone (Dexa, n=4). Significantly altered cell types between mild and severe are highlighted in gray, and those altered between severe +/- dexamethasone are marked by red arrows. **(D)** Cell types significantly altered by dexamethasone treatment in severe COVID-19 patients. Bars represent means ± SD. In **(A, D)**, each symbol represents an individual patient or healthy donor. Dexa = dexamethasone. Significance was determined by unpaired t-test and by Mann-Whitney rank sum test where samples failed normality. The abundance of the different immune cell types (at the RNA level) in the various patient cohorts was calculated as log_2_ cell type scores. The cell scores for a specific cell type can only be compared between two groups (such as mild vs. severe COVID-19) but do not support claims that a cell type is more abundant than another cell type within the same group.

Experiments were performed to determine whether the alterations in the host immune response to SARS-CoV-2 are related to disease severity and also to dexamethasone treatment. To that end, we generated transcriptomic data from PBMCs obtained from the above-mentioned COVID-19 patient cohorts and healthy controls, using the NanoString Host-Response panel. Clustering analysis of our transcriptomic datasets indicates that the PBMCs from COVID-19 patients (irrespective of disease severeity and dexamethasone treatment) group separately as compared to healthy individuals (**Figure 1A**). Furthermore, we also observed that most dexamethasone-treated severe COVID-19 patients and mild patients were clustered separately from the severe COVID-19 patients who did not receive the drug, indicating that disease severity as well as dexamethasone treatment could be a source of variation in our datasets. We then profiled the relative abundance of immune cell populations among the patient cohorts. We found an increase in CD56^dim^NK cells, suggesting increased cytotoxicity in mild patients compared to healthy donors (**Figure 1B**). We also detected a significant increase in exhausted CD8^+^ T cells in mild patients as compared to healthy individuals, while NK, CD8^+^ T, and Th1 cells were reduced, all of which can lead to ineffective pathogen clearance (**Figure 1B**). Compared to mild disease, individuals with severe COVID-19 showed fewer CD56^dim^NK cells, whereas patients with severe COVID-19 who received dexamethasone displayed decreased abundance of cytotoxic, CD56^dim^NK, Th1, exhausted CD8^+^, and mast cell populations (**Figures 1C, D**). Collectively, these data suggest that dexamethasone may exacerbate the reduction of CD56^dim^NK cells that occurs with disease severity, while controlling the exhausted T cells that were already increased in patients with mild disease.

**Figure 2:**
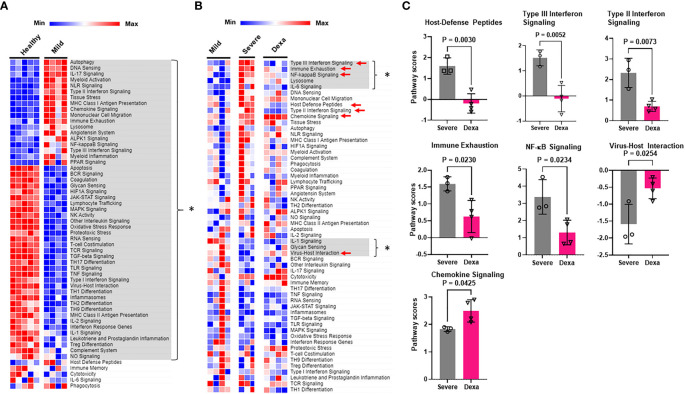
Immune signaling pathways altered by COVID-19 and by dexamethasone. **(A)** Heatmap depicting immune signaling pathway scores in PBMCs from healthy donors (n=5) and COVID-19 patients with mild disease (n=4). Significantly altered pathways are highlighted in gray and represented by * **(B)** Heatmap depicting immune signaling pathway scores in PBMCs from patients with mild (n=4), severe COVID-19 (Severe, n=3), and severe COVID-19 + dexamethasone (Dexa, n=4). Significantly altered pathways between mild and severe groups are highlighted in gray and represented by *, and those altered between severe +/- dexamethasone are marked by red arrows. **(C)** Pathways significantly altered by dexamethasone treatment in severe COVID-19 patients. Bars represent means ± SD, and each symbol represents an individual. Significance determined by unpaired t-test.

To elucidate the mechanisms by which SARS-CoV-2 alters immune cell functionality, we analyzed the immune pathways that were differentially expressed in our COVID-19 patient cohorts. Pathway analysis revealed significant alterations in multiple pathways responsible for innate immune cell activation, immune cell exhaustion, adaptive host immune responses as well as inflammatory and interferon responses in patients with mild COVID-19, as compared to healthy controls (**Figure 2A**). However, the primary focus of our study was to determine which pathways were enriched in severe COVID-19 patients compared to mild disease, and investigate if any of these enriched pathways were altered by dexamethasone treatment. In severe disease we observed an increase in immune exhaustion and an enhanced innate cell-driven inflammatory response. Specifically, we saw an increase in NF-κB, and IL-6 pathways. We also saw upregulation of the Type III interferon response pathways, while IL-1 signaling and virus-host interactions were significantly reduced (**Figure 2B**). In contrast, PBMCs from patients with severe COVID-19 who received dexamethasone, had a signaling pathway profile more similar to that of mild patients. This included reduced Type III interferon signaling, immune exhaustion, and NF-κB signaling pathways and increased virus-host interaction in severe patients treated with dexamethasone. Additionally, severe patients with dexamethasone showed reduced host-defense peptides, Type II interferon and chemokine signaling pathways (**Figures 2B, C**).

**Figure 3:**
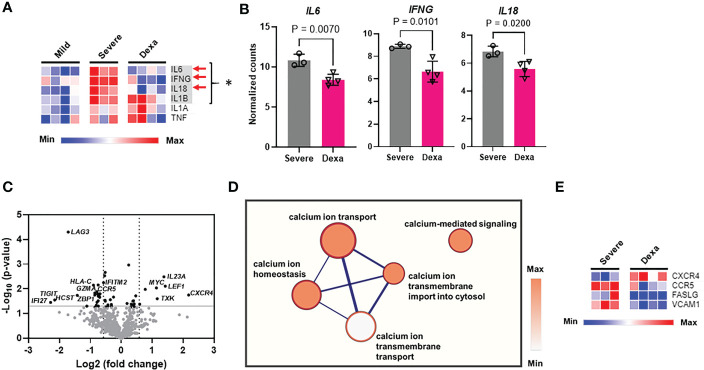
Cytokine storm gene expression, differential gene expression, and calcium signaling pathways altered by dexamethasone in severe COVID-19. **(A)** Heatmap depicting expression levels (determined as normalized RNA counts by NanoString) of genes involved in cytokine storm production in PBMCs from patients with mild (n=4), severe COVID-19 (Severe, n=3), and severe COVID-19 treated with dexamethasone (Dexa, n=4). Significantly altered genes between mild and severe groups are highlighted in gray and represented by *, and those altered between severe +/- dexamethasone are marked by red arrows. **(B)** Cytokine storm genes significantly altered by dexamethasone treatment in severe COVID-19 patients. Bars represent means ± SD, and each symbol represents an individual. Significance in **(A, B)** was determined by unpaired t-test. **(C)** Volcano plot showing differentially expressed genes (P<0.05 with unpaired t test, -log_10_Pvalue > 1.3, fold change>1.5) altered by dexamethasone in severe COVID-19. Top differentially expressed genes are labeled. **(D)** Correlation network analysis for calcium signaling-related pathways that were decreased by dexamethasone in severe COVID-19. The cluster of nodes that represent the transmembrane calcium ion transport are grouped together in the outer rectangle. Thicker edges (blue lines) indicate the number of genes shared between the nodes, whereas the intensity of the node fill color indicates the enrichment of genes in that specific process. **(E)** Heatmap depicting expression levels (determined as normalized RNA counts by NanoString) of significantly altered genes, that were involved in the calcium signaling-related pathways (shown in Panel **D**) in PBMCs from patients with severe COVID-19 (Severe, n=3), and severe COVID-19 treated with dexamethasone (Dexa, n=4). Further details and p-values for these genes are presented in **Table S5**. Significance determined by unpaired t-test.

We then identified the differentially expressed genes in severe COVID-19 patients with and without dexamethasone treatment. Dexamethasone resulted in differential expression of several genes involved in immune response of host to viruses, including genes relevant to cytokine storm. Cytokine storm is associated with ARDS that is the leading cause of mechanical ventilation in severe COVID-19, and these patients benefit from dexamethasone which acts by abrogating the cytokine storm, thus preventing patient mortality (3, 4, 8). We therefore compared the expression of the genes mediating cytokine storm in our patient cohorts and observed that, compared to individuals with mild COVID-19, patients with severe illness showed a significant increase in *IL6, IFNG, IL18*, and *IL1B*, while dexamethasone treatment decreased the abundance of *IL6, IFNG* and *IL18* (**Figures 3A, B**) (30). Other differentially expressed genes in severe COVID-19 patients after dexamethasone treatment are presented as a volcano plot in **Figure 3C** and are listed in **Table 1**. We first looked at the differentially expressed genes associated with the anti-viral immune response and inflammatory pathways that were significantly altered by dexamethasone in severe COVID-19 (shown in **Figure 2C**). Within these altered pathways, the most relevant genes downregulated by dexamethasone included: *TIGIT*, *LAG3*, *PDCD1*, *CTLA4*, *EOMES*, and *CD86* (immune exhaustion), *FASLG* (immune exhaustion and NF-κB signaling)*, VCAM1* (NF-κB and Type II interferon signaling); *IFNG, IRF7*, and *HLA-C* (Type II interferon signaling); *CCR5* (virus-host interaction and chemokine signaling); *TMPRSS2* (virus-host interaction); *IFNL1* and *IL10RB* (Type III interferon signaling); and *CD40LG* (NF-κB signaling), while the most relevant genes upregulated by dexamethasone included: *CXCR4* (chemokine signaling and virus-host interaction); *UBA52* and *CUL1* (NF-κB signaling). Furthermore, as shown in **Table 1**, in severe COVID-19 patients, dexamethasone treatment downregulated genes belonging to inflammatory and interferon signaling pathways that have been purported to contribute to COVID-19 disease severity which included type I interferon signaling (*IFI27*, *IFNB1*, *IFITM2*, *IFNA2*, *IRF7*, *ISG15*), inflammatory JAK-STAT and MAPK signaling (*IFNB1*, *IFNL1*, *IFITM2*, *IFITM3*, *IFNG*, *IL11RA*), innate immune signaling pathways such as NOD (nucleotide-binding and oligomerization domain)-like receptor (NLR) signaling (*IFNB*1*, IL6*, *IFNA2*, *IRF7*, *IL18*, *IF116*) and pathways influencing T cell receptor stimulation and NK cell activity (6, 31). Additionally, we observed that dexamethasone reduced the abundance of genes related to cytotoxicity (*GZMA*) and inflammatory cell death/PANoptosis (*ZBP1*) (31, 32). Overall, our immune profiling transcriptomic data highlight that PBMCs from patients with severe COVID-19 show a characteristic increase in immune exhaustion and various pro-inflammatory signaling pathways, as well as markers of cytokine storm and cytotoxicity, which are attenuated by dexamethasone administration.

**Table 1:** List of genes differentially expressed by dexamethasone treatment in severe COVID-19 patients.

Genes upregulated by dexamethasone in severely ill patients				
	gene	Full name	Pathways	P-value
1	*IFI27*	interferon alpha inducible protein 27	MAPK Signaling Type I Interferon Signaling	0.0001
2	*TIGIT*	T cell immunoreceptor with Ig and ITIM domains	**Immune Exhaustion** T-cell Costimulation	0.0008
3	*IFNB1*	interferon beta 1	DNA Sensing JAK-STAT Signaling NLR Signaling RNA sensing TNF Signaling Type I Interferon Signaling	0.0011
4	*LAG3*	lymphocyte activating 3	**Immune Exhaustion** MHC Class II Antigen Presentation T-Cell Costimulation	0.0012
5	*IFITM2*	interferon-induced transmembrane protein 2	MAPK Signaling Type I Interferon Signaling	0.0015
6	*PDCD1*	programmed cell death 1	**Immune Exhaustion** T-cell Costimulation TCR Signaling	0.0017
7	*IFNL1*	interferon, lambda 1	JAK-STAT Signaling Other Interleukin Signaling **Type III Interferon Signaling**	0.0024
8	*IFI6*	interferon alpha inducible protein 6	NLR Signaling DNA sensing	0.0025
9	*VCAM1*	vascular cell adhesion molecule 1	Lymphocyte Trafficking **NF-kB Signaling** Other Interleukin Signaling **Type II Interferon Signaling**	0.0029
10	*IFITM3*	interferon-induced transmembrane protein 3	MAPK Signaling Type I Interferon Signaling	0.0043
11	*IL6*	interleukin 6	IL-6 Signaling DNA Sensing HIF1A Signaling JAK-STAT Signaling Mononuclear Cell Migration Myeloid Activation NLR Signaling TH17 Differentiation Th2 Differentiation Tissue Stress TNF Signaling	0.0070
12	*RAC2*	Rac family small GTPase 2	BCR Signaling Lymphocyte Trafficking Myeloid Activation Myeloid Inflammation	0.0086
13	*IFNG*	interferon gamma	HIF1A Signaling JAK-STAT Signaling Myeloid Activation Other Interleukin Signaling TCR Signaling TGF-beta Signaling Th1 Differentiation **Type II Interferon Signaling**	0.0101
14	*IL10RB*	interleukin 10 receptor, beta	JAK-STAT Signaling Other Interleukin Signaling Treg Differentiation **Type III Interferon Signaling**	0.0116
15	*TMPRSS2*	transmembrane serine protease 2	**Virus-Host Interaction**	0.0141
16	*IFNA2*	interferon alpha 2	DNA Sensing JAK-STAT Signaling NLR Signaling RNA Sensing Type I Interferon Signaling	0.0157
17	*BCL3*	BCL3 transcription coactivator	TNF Signaling	0.0159
18	*LITAF*	lipopolysaccharide-induced TNF factor	Lysosome	0.0161
19	*IRF7*	interferon regulatory factor 7	DNA Sensing NLR Signaling RNA Sensing TLR Signaling Type I Interferon Signaling **Type II Interferon Signaling**	0.0179
20	*ISG15*	ISG15 ubiquitin-like modifier	Interferon Response Genes RNA Sensing Type I Interferon Signaling	0.0184
21	*CTLA4*	cytotoxic T-lymphocyte-associated protein 4	**Immune Exhaustion** T-cell Costimulation TCR Signaling	0.0186
22	*CD40LG*	CD40 ligand	**NF-kB Signaling** T-cell Costimulation TCR Signaling	0.0194
23	*IL18*	interleukin 18	DNA Sensing IL-1 Signaling Myeloid Activation NLR Signaling	0.0200
24	*TLR3*	toll-like receptor 3	DNA Sensing Myeloid Activation TLR Signaling	0.0211
25	*ITGB2*	integrin subunit beta 2	Complement System Lymphocyte Trafficking Myeloid Activation NK Activity Other Interleukin Signaling TLR Signaling	0.0211
26	*CCR5*	C-C motif chemokine receptor 5	**Chemokine Signaling** **Virus-Host Interaction**	0.0257
27	*GZMA*	granzyme A	Cytotoxicity	0.0273
28	*HLA-C*	major histocompatibility complex, class I, C	MHC Class I Antigen Presentation Myeloid Activation Type I Interferon Signaling **Type II Interferon Signaling**	0.0286
29	*IL18RAP*	interleukin 18 receptor accessory protein	IL-1 Signaling Myeloid Activation Oxidative Stress Response	0.0317
30	*CD86*	CD86 molecule	**Immune Exhaustion** Other Interleukin Signaling T-cell Costimulation	0.0331
31	*HCST*	hematopoietic cell signal transducer	NK Activity	0.0344
32	*SELENOS*	selenoprotein S	Oxidative Stress Response Proteotoxic Stress	0.0349
33	*MAF*	MAF bZIP transcription factor	Th2 Differentiation	0.0355
34	*FASLG*	Fas ligand (TNF superfamily, member 6)	Apoptosis Cytotoxicity **Immune Exhaustion** **NF-kB Signaling** Other Interleukin Signaling	0.0383
35	*IL11RA*	interleukin 11 receptor subunit alpha	IL-6 Signaling JAK-STAT Signaling	0.0399
36	*IFI16*	interferon alpha inducible protein 6	DNA Sensing NLR Signaling	0.0417
37	*CD45R0*	protein tyrosine phosphatase receptor type C	Immune Memory Myeloid Activation TCR Signaling	0.0508
38	*PRDM1*	PR/SET domain 1	BCR Signaling	0.0513
39	*EOMES*	eomesodermin	**Immune Exhaustion**	0.0522
40	*ZBP1*	Z-DNA binding protein 1	DNA Sensing	0.0523
1	*IL23A*	interleukin 23 subunit alpha	JAK-STAT Signaling Other Interleukin Signaling Th17 differentiation	0.0041
2	*SELL*	selectin L	Immune Memory Lymphocyte Trafficking Myeloid Activation	0.0077
3	*TXK*	TXK tyrosine kinase	Lymphocyte Trafficking Myeloid inflammation	0.0082
4	*HSP90AB1*	heat shock protein 90 alpha family class B member 1	Inflammasomes Myeloid Activation NLR Signaling Proteotoxic Stress Th17 Differentiation	0.0085
5	*LEF1*	lymphoid enhancer-binding factor 1	TCR Signaling	0.0096
6	*CXCR4*	C-X-C motif chemokine receptor 4	**Chemokine Signaling** Lymphocyte Trafficking **Virus-Host Interaction**	0.0102
7	*NLRP1*	NLR family pyrin domain containing 1	Inflammasomes NLR Signaling	0.0126
8	*STRAP*	serine/threonine kinase receptor-associated protein	TGF-β Signaling	0.0134
9	*MYC*	MYC proto-oncogene, bHLH transcription factor	Other Interleukin Signaling TGF-β Signaling	0.0159
10	*CDK4*	cyclin-dependent kinase 4	TCR Signaling	0.0194
11	*TNFRSF10B*	TNF receptor superfamily member 10b	Apoptosis Cytotoxicity	0.0262
12	*UBA52*	ubiquitin A-52 residue ribosomal protein fusion product 1	ALPK1 Signaling BCR Signaling DNA Sensing Glycan Sensing Interferon Response Genes **NF-κB Signaling** RNA Sensing TCR Signaling TGF-β Signaling TLR Signaling TNF Signaling	0.0275
13	*IKBKE*	inhibitor of nuclear factor kappa B kinase subunit epsilon	DNA Sensing NLR Signaling RNA Sensing TLR Signaling Type I Interferon Signaling	0.0313
14	*CUL1*	cullin 1	BCR Signaling Glycan Sensing **NF-kB Signaling** TCR Signaling TGF-β Signaling TLR Signaling	0.0520
15	*CD28*	CD28 molecule	T-cell Costimulation TCR Signaling	0.0543
16	*C1QBP*	complement C1q binding protein	Apoptosis Coagulation	0.0549

Differentially expressed genes were determined by comparing the normalized counts for all genes in NanoString Host Response Panel codeset between the severe COVID-19 (n=3) and severe COVID-19 treated with dexamethasone (n=4) patient groups. Pairwise comparisons were performed using unpaired t-tests. All of the genes that were significantly downregulated or upregulated by dexamethasone are presented in the table, along with their annotations. The pathway annotations for the individual differentially expressed genes are also shown below, and the pathways that are differentially expressed by dexamethasone (shown in **Figures 2B, C**) and shown in bold.

### 3.2 Impact of COVID-19 and dexamethasone on calcium-regulated pathways and Kv1.3 channels

Multiple signaling pathways that regulate the production of pro-inflammatory cytokines (including IFN-γ), chemokines and cytotoxic granules rely on cytosolic Ca^2+^, which regulates the activity and signaling pathways of multiple transcription factors including NF-AT and NF-κB (17, 33–35). Hence, we performed correlation network analysis to identify the biological processes associated with our gene sets and identified whether Ca^2+^ homeostasis processes were altered by treatment with dexamethasone. This analysis revealed that transmembrane Ca^2+^ ion transport-associated functions like Ca^2+^ ion transport into the cell and Ca^2+^-mediated signaling, were decreased by dexamethasone treatment (**Figure 3D**). We further queried the genes that constituted these altered calcium ion signaling-related pathways and found that *CXCR4*, *CCR5*, *FASLG* and *VCAM1* were differentially expressed (**Figure 3E**, **Table S5**). Since Ca^2+^ influx into cells is regulated by ion channels, we conducted experiments to investigate whether ion channels are involved in COVID-19 severity and the response to dexamethasone.

**Figure 4:**
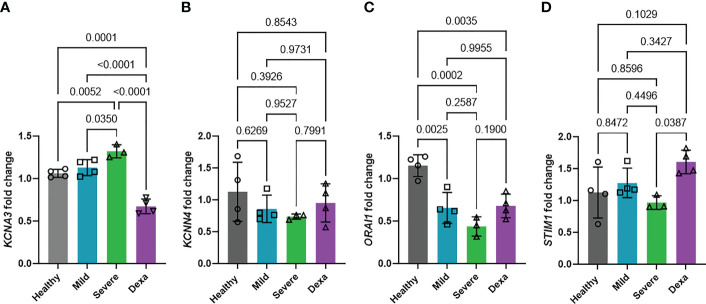
Changes in ion channel expression with disease progression and dexamethasone treatment. Fold change in mRNA abundance of Kv1.3 (*KCNA3*, **A**), KCa3.1 (*KCNN4*, **B**), Orai (*ORAI1*, **C**) and Stim1 (*STIM1*, **D**) in PBMCs from healthy donors (n=4), and patients with mild COVID-19 (n=4), severe COVID-19 (Severe, n=3), and severe COVID-19 + dexamethasone (Dexa, n=4) was determined by RT-qPCR. Data are normalized to healthy donor PBMCs. Each sample was run in triplicate. 18S rRNA was used as the housekeeping gene. Bars represent means ± SD, and each symbol represents an individual patient. Data were analyzed by one-way analysis of variance (ANOVA) (P < 0.001 in **A**, P = 0.4270 in **B**, P=0.0003 in **C,** and P=0.0414 in **D**), and *post hoc* testing was performed by the Holm-Sidak method.

The potassium channels Kv1.3 and KCa3.1, encoded by the *KCNA3* and *KCNN4* genes, respectively, and the CRAC channel (formed by two subunits encoded by the *ORAI1* and *STIM1* genes) are well-established regulators of Ca^2+^ entry into immune cells (16, 17). We investigated whether the *KCNA3*, *KCNN4*, *ORAI1*, and *STIM1* expression were altered in severe disease and determined the impact of dexamethasone treatment on channel expression. RT-qPCR experiments revealed that *KCNA3* expression was increased in severe COVID-19 as compared to mild COVID-19 patients and healthy controls (**Figure 4A**). Dexamethasone treatment decreased *KCNA3* expression to levels below those of healthy controls (**Figure 4A**). In contrast, neither disease severity nor dexamethasone treatment altered *KCNN4* expression (**Figure 4B**). *ORAI1* abundance was decreased in all of our COVID-19 patients compared to healthy controls (**Figure 4C**). However, there were no significant differences in *ORAI1* expression between mild and severe patients, and, additionally, dexamethasone treatment in severely ill patients did not affect *ORAI1* expression (**Figure 4C**). *STIM1* expression was not altered in mild or severe COVID-19 patients compared to healthy controls, but severe COVID-19 patients with dexamethasone displayed increased gene expression (**Figure 4D**). In summary, we demonstrate that *KCNA3* expression correlates with disease severity, and dexamethasone decreases the upregulation of *KCNA3* that is observed in severe COVID-19 to levels below that of healthy control PBMCs.

### 3.3 Corroborating evidence for a role of Kv1.3 in dexamethasone-mediated immunosuppressive effects

**Figure 5:**
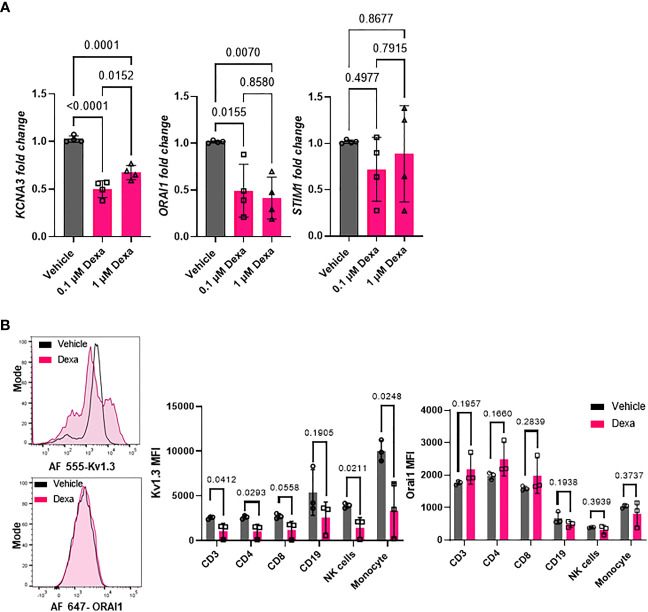
Effect of dexamethasone on Kv1.3 channel abundance. **(A)** Kv1.3 (*KCNA3*), Orai1 (*ORAI1*), and Stim1 (*STIM1*) mRNA expression in PBMCs from healthy donors (n=4) treated with 0.1 μM and 1μM dexamethasone or vehicle for 48 h was determined by RT-qPCR. Data were normalized to vehicle-treated control. Significance was determined by one-way analysis of variance (ANOVA) for *KCNA3* (P < 0.001), *ORAI1* (P=0.005), and by ANOVA on ranks for *STIM1* (p=0.630). *Post-hoc* testing was performed by the Holm-Sidak method. **(B)** Kv1.3 and Orai1 abundance in immune cell subsets from healthy donor PBMCs treated with 1 μM dexamethasone for 48 h. (Left) Representative flow cytometry histograms showing Kv1.3 (top) and Orai1 (bottom) abundance in the presence or absence of dexamethasone treatment in CD3^+^ T cell subsets. (Right) Mean fluorescence intensity (MFI) of Kv1.3 and Orai1 in immune cell subsets from three healthy donors treated *in vitro* with 1 μM dexamethasone or vehicle for 48 h. Significance determined by paired t-test. means ± SD, and each symbol represents an individual donor. .

Kv1.3 channels regulate cytokine and chemokine production as well as other immune cell functions, several of which are altered by dexamethasone treatment in severe COVID-19 patients (**Figure 2**) (15, 16). We conducted *in vitro* experiments in healthy donor PBMCs to verify our findings on altered *KCNA3*, *ORAI1*, and *STIM1* gene expression in dexamethasone-treated COVID-19 patients and confirmed that *in vitro* treatment with dexamethasone altered the abundance of these ion channel encoding genes. We isolated PBMCs from healthy individuals and exposed them to 0.1 and 1 μM dexamethasone for 24 and 48 h, while vehicle-treated PBMCs were used as controls. These concentrations were chosen to reflect the plasma concentrations of 23-72 nM reported in humans treated with dexamethasone at steady state (36). *In vitro* administration of dexamethasone did not affect cell viability of PBMCs (**Figure S1**). Dexamethasone treatment significantly decreased *KCNA3* abundance as compared to vehicle-treated control at 24 h (**Figure S2**) and 48 h (**Figure 5A**). *ORAI1* expression was not altered at 24 h (**Figure S2**) but was decreased at 48 h (**Figure 5A**). *STIM1* was not altered at either 24 h (**Figure S2**) or 48 h after dexamethasone (**Figure 5A**).

To determine whether *in vitro* treatment of healthy donor PBMCs with dexamethasone for 48 hours altered Kv1.3 and Orai1 protein levels, we performed flow cytometry. We designed a multicolor flow cytometry panel for leukocytes and mononuclear cells (see Materials and Methods) to assess Kv1.3 and Orai1 protein abundance in various immune cell subsets, as defined by gating strategies presented in **Figure S3**. We observed that dexamethasone significantly reduced Kv1.3 protein abundance in CD3^+^ T cells, CD4^+^ T cells, CD56^dim^NK cells, and monocytes as compared to vehicle-treated controls after 48 h treatment (**Figure 5B**). Furthermore, in the CD8^+^ T cell subset, we observed a trend towards decreased Kv1.3 abundance (p=0.0558, **Figure 5B**), while dexamethasone did not alter Kv1.3 abundance in the CD19^+^ B cell subset (**Figure 5B**). Dexamethasone did not alter the percentage of Kv1.3^+^ cells (**Figure S4**) and Orai1 protein abundance in each immune cell subset (**Figure 5B**).

**Figure 6:**
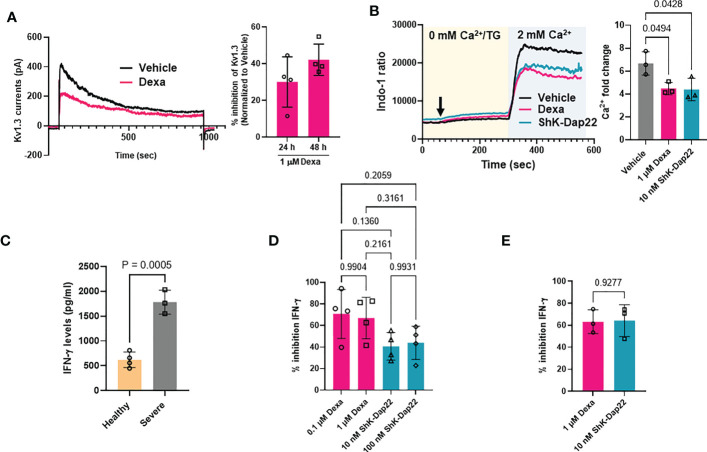
Effect of dexamethasone on Kv1.3 channel function, Ca^2+^ signaling and cytokine production. **(A)** Inhibition of Kv1.3 currents in CD8^+^ T cells treated with 1 μM dexamethasone (Dexa) for 24 and 48 h. Representative Kv1.3 currents are shown on the left, and percentage inhibition of Kv1.3 currents by dexamethasone in n=4 donors is shown on the right. **(B)** Representative Ca^2+^ response (shown as a ratio of Indo-1 fluorescence at 400 and 480 nm) recorded in activated healthy donor CD8^+^ T cells treated with either 1μM dexamethasone, 10 nM ShK-Dap22, or vehicle for 48 h are shown on the left. Cells were loaded with Indo-1 ratiometric dye, and the fluorescence was recorded by flow cytometry. Indo-1 loaded cells were first exposed to thapsigargin (arrow) in 0 mM Ca^2+^, followed by 2 mM Ca^2+^, which yields a rapid influx of Ca^2+^ (see Materials and Methods). Data are representative of independent experiments performed in CD8^+^ T cells isolated from n=3 healthy donors. The average fold change in peak Ca^2+^ levels in n = 3 healthy donors are shown on the right. Significance was determined by one-way analysis of variance (ANOVA, p=0.0309), and *post hoc* testing was performed by Tukey’s test. **(C)** IFN-γ release determined by ELISA in the supernatant of activated CD8^+^ T cells from n=4 healthy donors and n=3 severe COVID-19 patients. Significance was determined by unpaired t test **(D)** Percent inhibition of IFN-γ secretion as compared to vehicle treated cells after treatment of activated healthy donor (n=4) CD8^+^ T cells with either 0.1 μM and 1μM dexamethasone, 10 nM and 100 nM ShK-Dap22, or vehicle for 48 h. Significance was determined by one-way analysis of variance (ANOVA, p=0.9390). **(E)** Percent inhibition of IFN-γ secretion compared to vehicle treated cells after treatment of activated severe COVID-19 patient (n=3) CD8^+^ T cells with either 1μM dexamethasone, 10 nM ShK-Dap22, or vehicle for 48 h. Significance was determined by paired-t test. Activation of CD8^+^ T cells in panels **(B–E)** was done for 48 h with plate bound anti-CD3 and anti-CD28 antibodies. Bars represent means ± SD, and each symbol represents an individual.

We then conducted functional studies to determine whether the decreased abundance of *KCNA3* by dexamethasone translated into an actual reduction of functional Kv1.3 channels and inhibition of effector functions. We conducted these experiments in CD8^+^ T cells that are critical to mounting an effective antiviral immune response in severe COVID-19 (2). We thus exposed CD8^+^ T cells from healthy donors to dexamethasone (0.1 μM and 1 μM) for 48 h (controls were treated with DMSO, vehicle) and performed electrophysiological experiments to assess whether the decreased Kv1.3 expression by dexamethasone in CD8^+^ T cells correlated with reduced Kv1.3 channel function. Kv1.3 activity (defined by the Kv1.3 peak current) was significantly inhibited in dexamethasone-treated CD8^+^ T cells as compared to vehicle-treated controls (**Figure 6A**, **Table S6**). This inhibition of Kv1.3 currents by dexamethasone was similar to that induced by ShK-Dap22, a specific Kv1.3 channel blocker, at concentrations that fully block the channel activity (**Figure S5**). On exposure to dexamethasone, there was no change in cell capacitance, a measure of cell size, and an indication of the activation state of these cells (**Table S6**) (19).

It is well-documented that a reduction in Kv1.3 activity leads to a reduction in the Ca^2+^ influx in T cells (15–17, 29). Thus, we measured the Ca^2+^ response of activated CD8^+^ T cells isolated from healthy donors that were exposed to either dexamethasone or ShK-Dap22. Representative recordings of intracellular Ca^2+^ levels in dexamethasone, ShK-Dap22 and vehicle treated CD8^+^ T cells from the same individual are presented in **Figure 6B** (left). Using the Ca^2+^ add-back method, the endoplasmic reticulum (ER) Ca^2+^ stores are emptied by adding TG (a SERCA pump inhibitor) in Ca^2+^-free medium, which opens the CRAC channels and results in Ca^2+^ influx upon readdition of extracellular Ca^2+^ in a T cell receptor (TCR) independent and ion channel dependent manner (29, 37). The Ca^2+^ recordings show that, compared to the vehicle-treated controls, dexamethasone and ShK-Dap22 reduced the Ca^2+^ influx. Dexamethasone significantly reduced the peak Ca^2+^ levels achieved during re-introduction of Ca^2+^ in the media by 33% (n = 3 healthy donors) (**Figure 6B**, right). We observed a similar reduction in peak Ca^2+^ with ShK-Dap22 (34%) (**Figure 6B**). Comparably, the AUC was deceased by ~35% in both treatment groups (**Figure S6**). These data show that the inhibition of Kv1.3 channels by dexamethasone contributes to the suppression of Ca^2+^ signaling. We further investigated the effect of dexamethasone on IFN-γ production, downstream to ion-channel mediated Ca^2+^ signaling, in both severe COVID-19 patients and healthy donors CD8^+^ T cells (16, 18, 22). We observed a 3-fold increase in IFN-γ production in activated CD8^+^ T cells from severe COVID-19 patients as compared to healthy individuals (**Figure 6C**) as expected as these patients underwent a cytokine storm (**Figure 3A**). The IFN-γ production was significantly reduced by dexamethasone and Kv1.3 blockade in CD8^+^ T cells from healthy donors (**Figure 6D**) and severely ill patients (**Figure 6E**). These data show that the decrease in functional Kv1.3 channels and Ca^2+^ signaling brought about by dexamethasone in CD8^+^ T cells from healthy volunteers and severely ill COVID-19 patients contributes to their attenuated effector functions, thereby establishing a new mechanism for the beneficial effects of dexamethasone in severe COVID-19.

## 4 Discussion

Herein, we report transcriptomic and functional studies that not only identify the immune and molecular signaling mechanisms that are altered in severe COVID-19, but also may account for some of the beneficial effects of dexamethasone in severely ill COVID-19 patients. Comparative transcriptomic analysis in PBMCs from patients with mild and severe COVID-19 disease demonstrated that severe illness altered immune pathways involved in inflammation including cytokine release, T cell exhaustion, interferon signaling and anti-viral immunity. Furthermore, we provide evidence that dexamethasone treatment of severe patients reversed these changes. These modified immune responses associated with disease progression and the effect of dexamethasone converge on Kv1.3 channels that regulate Ca^2+^ signaling in immune cells.

Several studies in COVID-19 patients have highlighted the contribution of abnormal host immune responses to disease pathogenesis and progression (3, 6, 31, 38–40). While little is known about the molecular mechanisms regulating mild versus severe disease, there is consensus that the pathogenesis of SARS-CoV-2 pneumonia is due to aberrant local and systemic inflammatory response elicited by the innate and adaptive immune cells to clear the virus (3, 31). While, in our study, early disease (mild patients) showed a profound alteration of multiple inflammatory pathways (50/55 pathways in our NanoString transcriptomic panel), we observed that disease severity in comparison, is accompanied by the alteration of relatively few key pathways as compared to mild patients (8/55 pathways). In agreement with published data, we observed that severe COVID-19 increased immune exhaustion, lysosome pathway, and Type III interferon, combined with elevated NF-κB and IL-6 signaling leading to increased levels of circulating pro-inflammatory cytokines, or “cytokine storm” (4, 6, 40–42). The current standard of care for the cytokine storm in severe COVID-19 focuses on conventional anti-viral drugs like remdesivir, to block viral entry, and dexamethasone, to inhibit the immune system (43). The primary benefit of dexamethasone in severe COVID-19 is thought to be suppression of cytokine storm (12). An intricate interplay between immune cell types, inflammatory cytokines and immune cell signaling pathways is responsible for the cytokine storm in severe COVID-19 patients (4). While conventional anti-viral therapeutics have limited efficacy, dexamethasone successfully improved the clinical outcomes, and most importantly prevented patient mortality in these patients (5, 8). While it is well-known that dexamethasone is anti-inflammatory in function, the mechanisms underlying its beneficial effects in severe COVID-19 have not been defined. Our transcriptomic analysis revealed the immune and molecular mechanisms mediating the effect of dexamethasone in severe COVID-19. Our approach in evaluating the same was to highlight the inflammatory and immune pathways and genes that were differentially expressed by dexamethasone in severe COVID-19 patients, and more importantly to investigate whether this altered transcriptomic profile was similar to mild COVID-19 patients. We assume that this correction indicated a “beneficial effect” of dexamethasone (albeit some of the immune alterations in severe patients may be adaptive in nature and not pathogenic). Leukopenia is characteristic of severe COVID-19 (3, 6, 44). Severe COVID-19 patients show depletion of NK, CD3, and CD8 cells, which impairs the immune response against the virus and contribute to further progression of the disease severity (3, 44, 45). However, the reduction of these cells in severe COVID-19 is a double-edged sword as, while they are needed to fight the virus, they also contribute to tissue inflammation. In accordance with published findings, we observed a reduction in CD56^dim^NK cell populations in the severe COVID-19 group. This cell population was further reduced in our dexamethasone-treated population, along with a reduction in cytotoxic cells, mast cells, Th1 cells and exhausted CD8^+^ T cells (6). Dexamethasone is known to reduce the number of T cells and also inhibit Th1 cell-mediated activation of NK cells and macrophages (10).

Cytokine storm is characterized by the overproduction of IL-1, IL-6, IL-18, IFN-γ and TNF-α (4, 30). We observed that patients with severe illness displayed an increased abundance of the genes encoding for IL-1 (*IL1B*), IL-6, IL-18 and IFN-γ, whereas dexamethasone-treatment decreased genes encoding for IL-6, IFN-γ, and IL-18. Higher levels of IL-6 in severely ill individuals correlate with higher mortality and thus a reduction in IL-6 by dexamethasone could contribute to improving the disease outcomes for severely ill patients (3, 4, 6, 31, 46). The pro- inflammatory cytokines that define the cytokine storm are induced by the pattern recognition receptors (PRRs) that sense the viral molecules, and are expressed by the innate immune cells. A crucial signaling event mediating this pro-inflammatory cytokine induction by PRRs is activation of the NF-κB pathway (4, 5, 31, 47). In accordance with published reports, we observed that NF-kB signaling pathway was upregulated in severe disease (6, 31). However, we observed decreased NF-kB signaling post-dexamethasone treatment. We also observed that severe COVID-19 patients treated with dexamethasone had decreased *ZBP1* expression. *ZBP1* is a Z-RNA–sensing PRR which promotes NF-κB–driven proinflammatory cytokine expression in response to SARS-CoV-2 infection and is strongly induced by type I and type III IFNs (31, 32). Interestingly, single cell transcriptomic studies recently showed that the expression of *ZBP1* was increased in immune cell subsets from patients with COVID-19 as compared to healthy controls, and this increased expression correlated with increased patient mortality (32). Moreover, in a preclinical mouse study, increased *ZBP1* expression in mice infected with SARS-CoV-2 induced inflammatory cell death and limited the efficacy of IFN therapy, a result that could be reversed by genetic deletion of *ZBP1* (32). We can thus speculate that reduction in ZBP1 by dexamethasone may similarly limit the inflammatory cell death in severe COVID-19 and may facilitate an improved therapeutic response.

IFN-γ, a type II interferon secreted by NK cells and T cells, is instead reduced by dexamethasone together with IL-18 (a cytokine which stimulates IFN-γ production) (4). IFN-γ is critical for host-immune response against COVID-19 (4, 48). However, based on current literature, the role of IFN-γ in COVID-19 is ambiguous. Commensurate with our findings, IFN-γ levels are increased in the serum of COVID-19 patients, and also in healthy donor PBMCs infected ex-vivo with SARS-CoV-2 (6, 32, 38, 49). While IFN-γ production can be beneficial as it decreases viral loads and enhances T cell cytotoxicity, persistent high levels of IFN-γ in cytokine storm however potentiate hyperinflammation by recruiting macrophages and leads to lung injury and ARDS in severely ill patients (50). Type III interferon pathway is also upregulated in severe COVID-19 patients. Others have shown instead that type III interferons are attenuated in severe COVID-19 disease (51). This discrepancy may be due to the gene analytical method used (measurement of secreted cytokines vs measuring of transcriptomic changes). Similarly, while several studies report delayed or attenuated type I interferon signature in severe disease, we did not see any change in the type I interferon signature with severe disease (6). Type I interferon signaling is one of the earliest protective responses against SARS-CoV-2 virus (5, 6, 31). Type I and type III interferons induce similar sets of interferon stimulated genes (ISGs) and activate overlapping signaling pathways (7, 52). Neutrophils from severe COVID-19 patients treated with dexamethasone downregulated ISGs (30). We report that dexamethasone treatment downregulated the type III interferon pathway, along with IFNΛ1 expression, but not type I interferon pathway, suggesting that this could be another mechanism by which dexamethasone inhibits proinflammatory cytokine production in severe disease. Furthermore, dexamethasone inhibited genes belonging to the NLR signaling pathway, which is responsible for activating the inflammasome and production of pro-inflammatory cytokines and pyroptotic cell death (31, 47). Thus, this reduction in inflammatory cytokines, inflammatory and interferon signaling pathways by dexamethasone can be an advantage in severe COVID-19.

Along with hyperinflammation in severe COVID-19, there is overwhelming evidence in the literature pointing towards cytotoxic cell immunosuppression due to upregulation of immune exhaustion markers (PD-1, TIGIT, Tim-3, CTLA-4) as an alternative mechanism to severe disease pathogenesis (6, 40, 53–56). Accordingly, we observed an increased immune exhaustion signature in severe COVID-19. While it has been reported that dexamethasone upregulated CTLA-4 abundance in lymphocytes *in vitro*, in our cohort of severe COVID-19 patients, dexamethasone suppressed the gene encoding for CTLA-4, as well as other exhaustion signature genes TIGIT, LAG3, PD1, FASL, Eomesodermin and CD86 (40, 57, 58). Interestingly, we also observed that dexamethasone decreased the abundance of TMPRSS2, a serine protease which facilitates SARS-CoV-2 entry (59). In summary, our transcriptomic findings show that dexamethasone corrects some of the pathways that are altered in severe COVID-19 as compared to mild patients, namely it reduces immune exhaustion, type III interferon and NF-κB signaling, while restoring virus-host interactions. The transcriptomic changes we report in severe COVID-19 patients as a consequence of dexamethasone treatment were associated with changes in Ca^2+^ signaling and Ca^2+^ transport functions, thus implicating ion channels in the development of severe disease and the response to dexamethasone.

We showed that Kv1.3 expression was significantly increased in PBMCs from severe COVID-19 patients raising the possibility that upregulation of Kv1.3 could contribute to the hyperactivity of immune cells and increased production of IFN-γ by CD8^+^ T cells in severe COVID-19. Indeed, we reported here that INF-γ release is 3-fold higher in severe COVID-19 patients as compared to healthy donor. Although it is well established that Kv1.3 channels are positive regulators of T, B, NK, and macrophage function (60–62), this is the first report to our knowledge, of data supporting a role for Kv1.3 in the pathogenesis of severe COVID-19. Since Kv1.3 is overexpressed in effector memory T cells and we used a mixed population of PBMCs, it is possible that the differences we observed in Kv1.3 expression in severe patients compared to mild may be underestimated (15, 63, 64). In accordance with previously published reports, which show that dexamethasone inhibits Kv1.3 channels in Jurkat T and human macrophage cell lines, we observed that Kv1.3 expression was lower in PBMCs from dexamethasone-treated group (13, 23). Our *in vitro* studies supported this finding as dexamethasone decreased Kv1.3 expression in T and NK cells and monocytes but not B cells. Functional studies in CD8^+^ T cells, which provide a critical antiviral immune response in severe COVID-19, showed that the dexamethasone-induced changes in Kv1.3 gene expression translated into decreased channel activity, Ca^2+^ influx and production of IFN-γ strongly suggesting that Kv1.3 contributes to dexamethasone-mediated immunosuppression (2, 31, 65).

Our findings raise the prospect that Kv1.3 blockade could serve as a novel therapeutic approach in severe COVID-19. Kv1.3 blockers have shown benefits as immune-suppressive agents in autoimmune diseases (22, 66, 67). Dalazatide, a Kv1.3 channel inhibitor currently in clinical trials, has been reported to reduce inflammatory cytokines such as IFN-γ, IL-17, and TNF–α in CD4^+^ and CD8^+^ T cells from patients with systemic lupus erythematosus (SLE) (68). We have also shown that cell-targeted Kv1.3 knockdown reduced Ca^2+^ influx, CD40L and IFN-γ in memory T cells from SLE patients (18, 22, 29). The advantage of Kv1.3 blockers over dexamethasone resides in the possibility that, while the former may not induce a global immune suppression, they may still suppress the cytokine storm but leave the body able to clear acute infections. Indeed, Kv1.3 blockers target activated effector memory T cells which, when unregulated, contribute to damaging inflammation, while leaving naïve and central memory T cells able to provide an acute response to immunological challenges (KCa3.1 controls the function of these T cells) (15). Furthermore, the benefits of a Kv1.3 targeted therapy may not be limited to severe COVID-19, but also extended to “Long Covid” syndrome or post-acute sequelae of SARS-CoV-2 infection (PASC) as it could inhibit the chronic inflammation in the nervous system [Kv1.3 channels regulate microglia function (60, 62)] and pulmonary arteries that are associated with these long-forms of the disease that depend on memory T cells (3).

There are limitations to our study. First, this was a study performed on only a small patient cohort. With a disease as heterogenous as COVID 19, this warrants a conservative interpretation of the results (69). A study in a larger cohort could confirm our findings of borderline significance and elicit other important mechanisms. Second, the samples included in this study were procured at the beginning of the pandemic when the original Wuhan strain (Wuhan-Hu-1) was dominant, and vaccinations were not yet available. Further studies on PBMCs from patients infected with more recent strains of SARS-CoV-2 may confirm the pivotal role of Kv1.3 channels in the disease pathogenesis. Thirdly, these samples were not obtained from a clinical trial in which severe COVID-19 patients were randomized to dexamethasone-treated or control groups. As these samples were collected prior to RECOVERY, there could be a possible clinical bias in which patients who received empiric steroids were more ill. Moreover, we were not able to determine if the clinical outcomes in the dexamethasone-treated patients were better than untreated. To attempt to control for this, we calculated the Sequential Organ Failure Assessment (SOFA) scores, and the number of organ failures at the time of patient sampling was similar for both cohorts. Furthermore, while interpreting the results of this study, we should also take into account that other glucocorticoid receptor (GR) mediated immune suppressive mechanisms like upregulation of checkpoint inhibitors are also taking place. Nevertheless, our study not only convincingly demonstrates a mechanism for the therapeutic effect of dexamethasone that is consistent with the literature, but also underscores the potential benefits of a therapy targeting Kv1.3 channels in COVID-19. This treatment modality could be of benefit in other pathologies where a cytokine storm occurs as a result of other pathogenic microbial infections, therapies (iatrogenic), cancers, or autoimmunity.

## Data availability statement

The datasets presented in this study can be found in online repositories. The names of the repository/repositories and accession number(s) can be found below: GSE227341 (GEO).

## Ethics statement

The studies involving human participants were reviewed and approved by University of Cincinnati Institutional Review Board. The patients/participants provided their written informed consent to participate in this study.

## Author contributions

Conceptualization: AC and LC; Methodology: AC and LC; Formal Analysis: AC and LC; Investigation: AC, AA, VG, and MC; Resources: KH and MP-F; Visualization: AC, AA, and MC; Project administration: AC and LC; Supervision: LC; Writing – original draft: AC and LC; Writing – review and editing: AC and LC; Funding acquisition: LC. All authors discussed the results and commented on the manuscript. All authors contributed to the article and approved the submitted version.
