# Supplementary material for: Immune and ionic mechanisms mediating the effect of dexamethasone in severe COVID-19
Source: Front Immunol. 2023 Mar 24;14:1143350. doi: 10.3389/fimmu.2023.1143350 (PMC10080085; doi:10.3389/fimmu.2023.1143350)
Supplement: Supplementary file 1 [file DataSheet_1.docx]

**
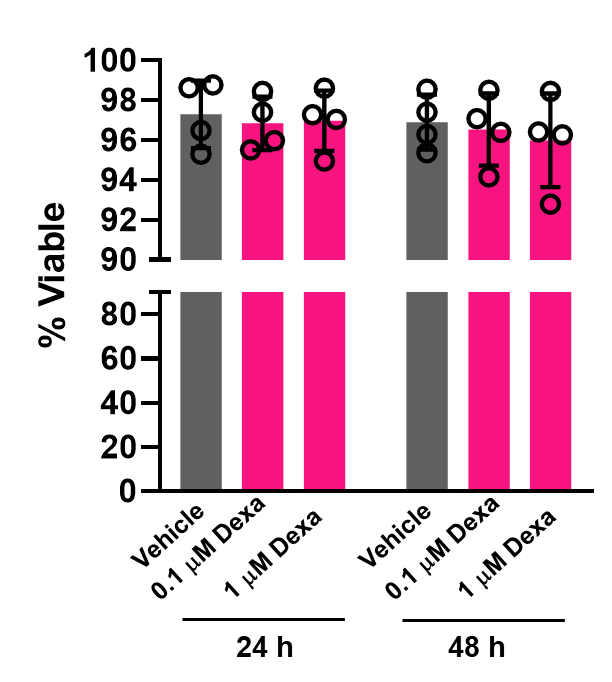
**

**Figure S1.** **Cell viability in healthy donor PBMCs treated with dexamethasone.**  Healthy donor PBMCs were treated with 0.1 μM and 1 μM dexamethasone for 24 h and 48 h and cell viability was determined by trypan blue exclusion which is shown on the y-axis as percent viable cells. Data presented as mean ± SD for n=4 healthy donor PBMCs treated with dexamethasone. Each symbol represents an individual donor.

**
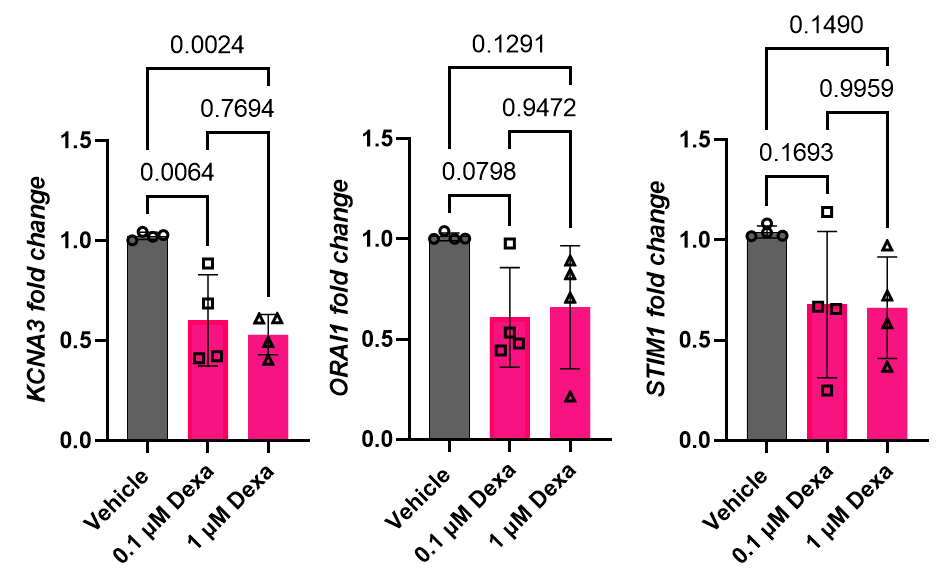
**

**Figure S2.** **Ion channel gene expression in healthy donor PBMCs after 24 h treatment with dexamethasone.** Healthy donor PBMCs (n=4) were treated with 0.1 μM and 1 μM dexamethasone or vehicle for 24 h, and Kv1.3 (*KCNA3*), Orai1 (*ORAI1*) and Stim1 (*STIM1*) mRNA expression was determined by RT-qPCR. Data normalized to vehicle-treated cells. Each sample was run in quadruplicate. 18S rRNA was used as housekeeping gene. Bars represent means ± SD, and each symbol represents an individual. Significance was determined by one-way ANOVA on ranks for *KCNA3* (P=0.011), and one-way ANOVA for *ORAI1* (P=0.005) and *STIM1* (p=0.630). Post-hoc testing was performed by Tukey’s test.

**
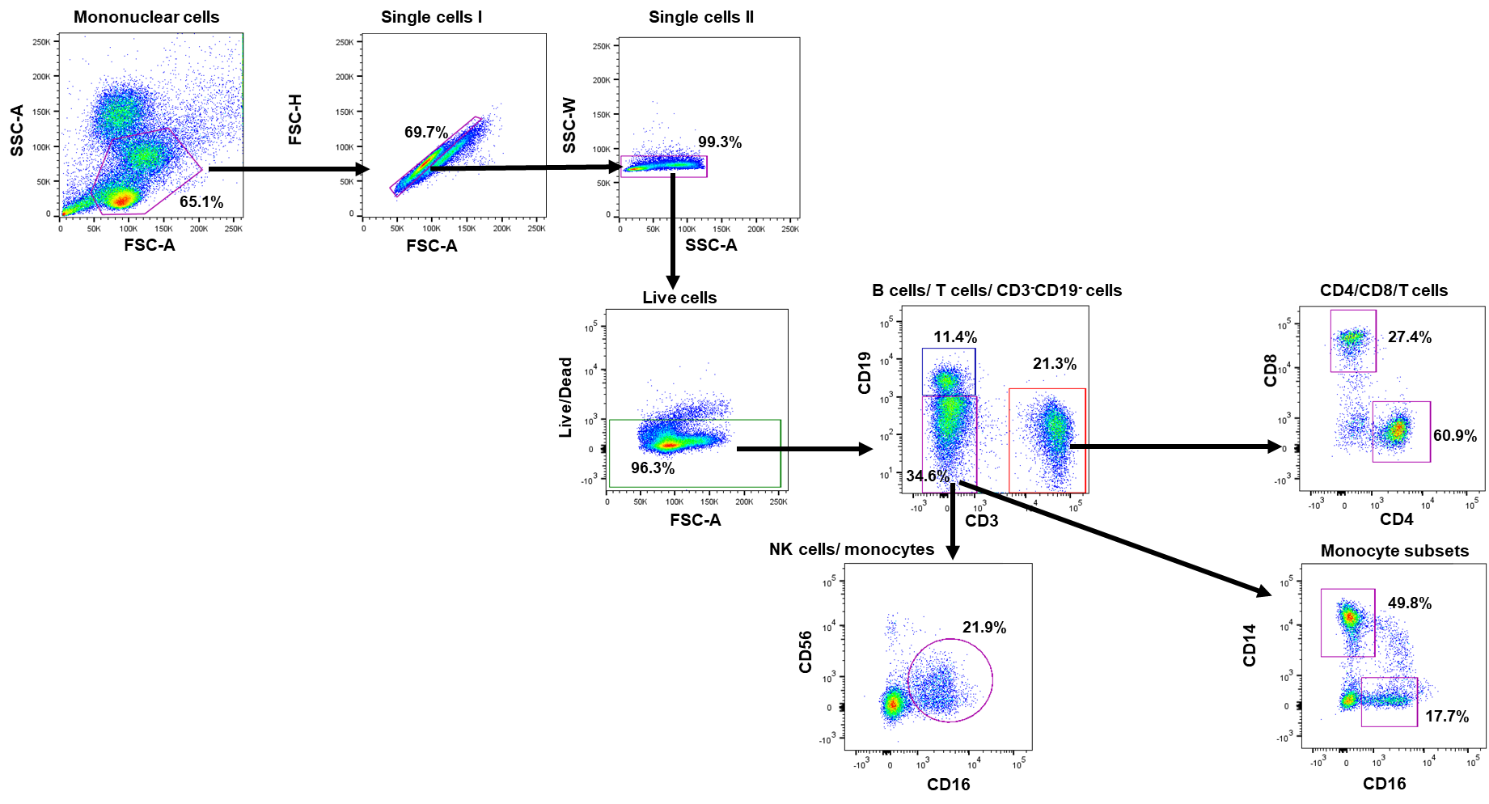
**

**Figure S3.** **Gating strategy for immune cell phenotyping flow cytometry experiments.** Representative gating to identify various immune cell phenotypes from PBMCs from a single healthy donor is shown here. Briefly, out of the total events acquired, we first gated the mononuclear cell popular, followed by single cells and live cells (gated as Zombie UV^+^). The live cell population was then gated as CD3-BV421^+^ T cells, CD19-APC-Cy7^+^ B cells or CD3^-^CD19^-^ population. The CD3^+^ T cells were further classified into CD4-BV605^+^ and CD8-BV711^+^ subpopulations. The CD56-PE-Dazzle 594^dim^ CD16^-^ NK cell population was identified within the CD3^-^CD19^-^ population. We then subdivided the monocyte subsets in the CD3^-^CD19^-^ population based on the expression of CD16-Alexa Flour 488 and CD14-BV510. In all of the immune cell subsets that were defined as shown, we then measured the Kv1.3 and Orai1 abundance.

**
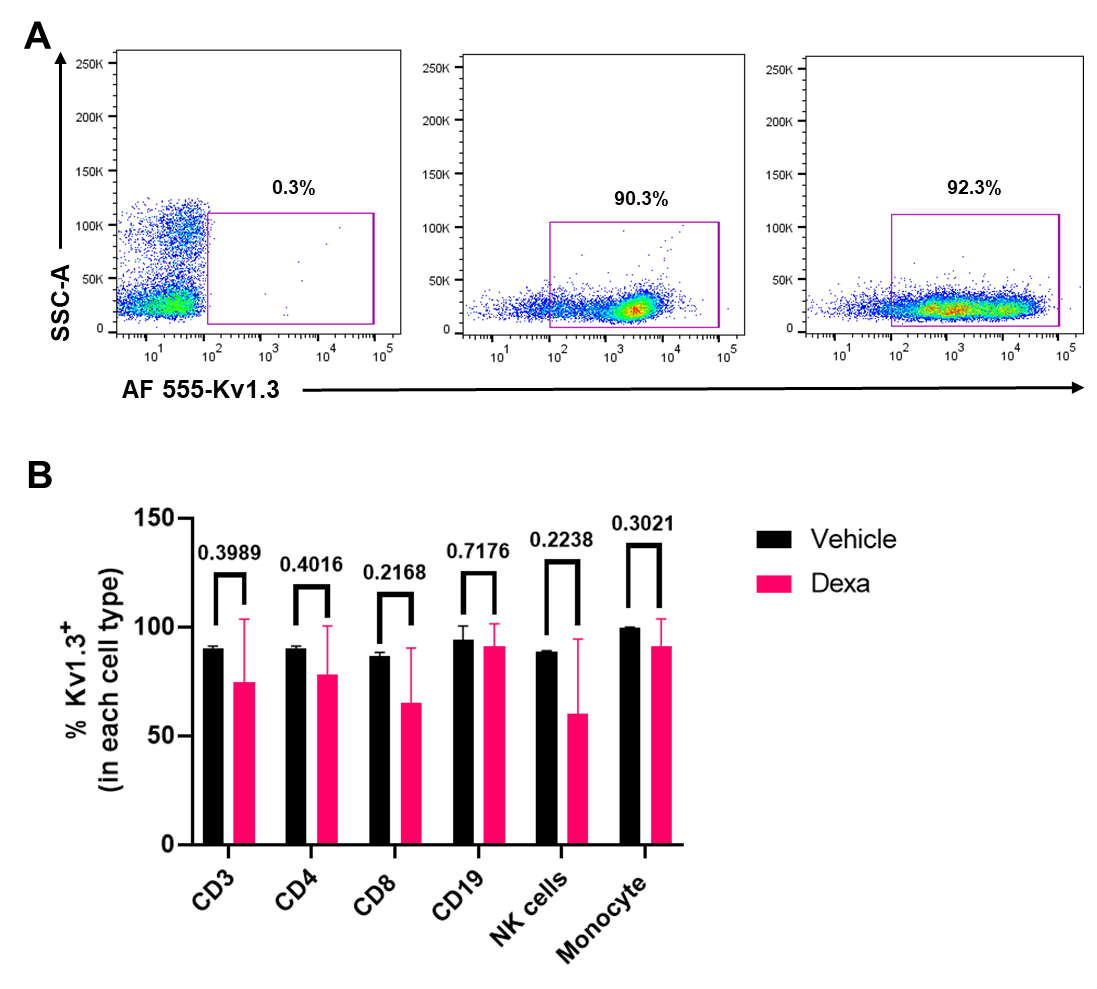
**

**Figure S4.** **Kv1.3 expression in healthy donor PBMCs treated with dexamethasone (A)** Representative flow cytometry plots gated on CD3^+^ T cell population in healthy donor PBMCs treated with 1 μM dexamethasone for 48 h showing Kv1.3 expression. **(B)** Quantification of Kv1.3 abundance in various immune cell subsets cells in three healthy donors treated with 1 μM dexamethasone for 48 h. Bars represent means ± SD. Significance determined by paired t-tests for each cell type.


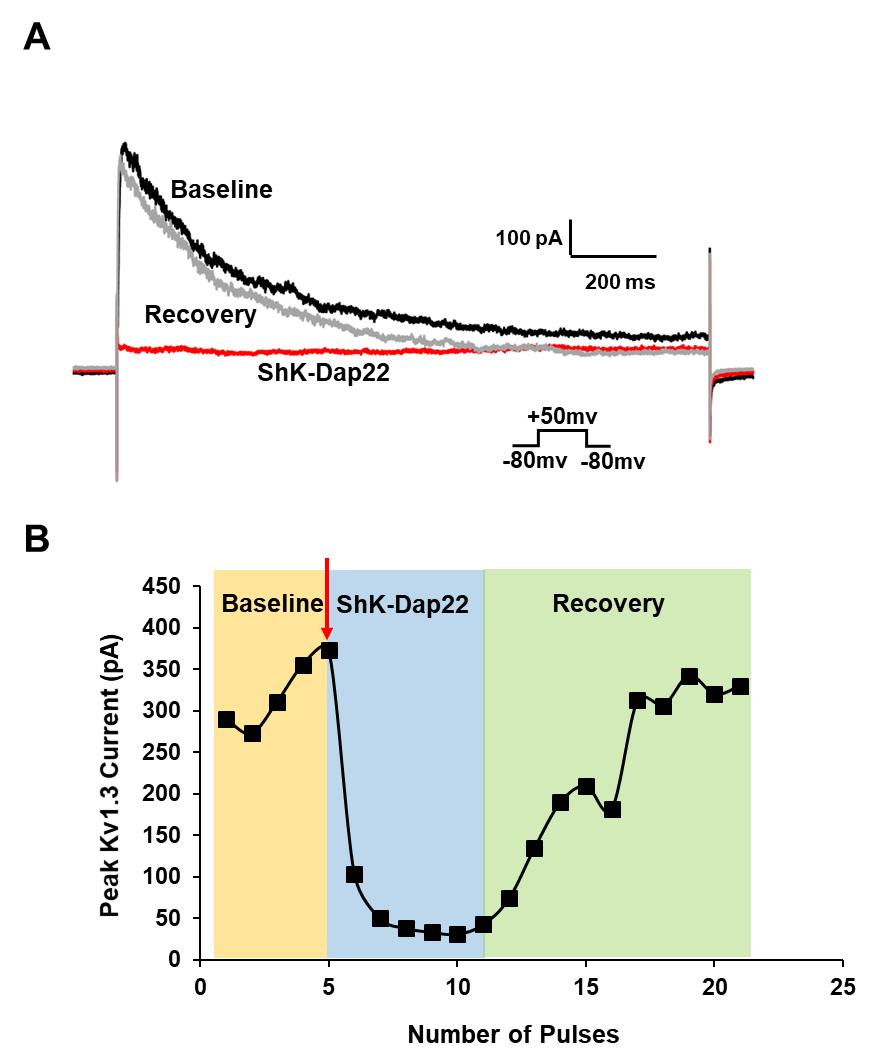


**Figure S5.** **Inhibition of Kv1.3 currents by ShK-Dap22 (A)** Representative Kv1.3 currents recorded in an activated CD8^+^ T cell (baseline), then in the presence of ShK-Dap22 (10 nM), followed by washout of the drug with external solution (recovery). **(B)** Representative experiment showing the time course of the change in the peak Kv1.3 currents from the baseline by addition of ShK-Dap22 (red arrow), followed by washout of the drug with the external solution (recovery).


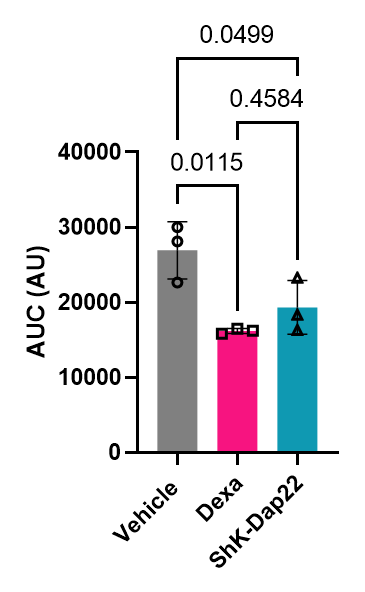


**Figure S6.** **Ca^2+^ influx in healthy donor CD8^+^ T cells treated with dexamethasone and ShK-Dap22.** The Area Under the Curve (AUC) post exposure to 2 mM Ca^2+^, which accounts for potential changes in the kinetics of Ca^2+^ fluxes (shown in Fig. 6B), was determined in healthy donor CD8^+^ T cells treated with either 1 μM dexamethasone (Dexa), 10 nM Shk-Dap22 or vehicle (control). Shown here are data for n=3 healthy donor CD8^+^ T cells that were activated for 48 h with plate-bound anti-CD3/CD28 antibodies. Significance was determined by one-way analysis of variance (ANOVA, p=0.0124), and post hoc testing was performed by Tukey’s test. Bars represent means ± SD, and each symbol represents an individual.

**Table S1.** **Demographics and co-morbidities of study patients**. Demographics and comorbidities for all patients with mild COVID-19 (n=4), severe COVID-19 (Severe, n=3) and severe COVID-19 patients treated with dexamethasone (Dexa, n=4) included in the study are summarized. All data were provided by the Center for Health Informatics, University of Cincinnati. BMI, body mass index; CAD, coronary artery disease; DM, diabetes mellitus; HF, heart failure, HTN, hypertension; N/A, data “not available” in the report.

| Patient ID | Cohort | Age | Gender | Race | BMI | Co-morbidities | | | | | | |
| --- | --- | --- | --- | --- | --- | --- | --- | --- | --- | --- | --- | --- |
|  |  |  |  |  |  | HTN | DM | Obesity | HF | CAD | Asthma | Cirrhosis |
| CCR0069 | Mild | 38 | Female | Hispanic | N/A | N | N | N | N | N | N | N |
| CCR0086 | Mild | 45 | Male | Hispanic | N/A | Y | Y | N | N | N | N | N |
| CCR0222 | Mild | 39 | Female | African  American | N/A | N | N | N | N | N | N | N |
| CCR0243 | Mild | 51 | Male | Caucasian | N/A | N | N | N | N | N | N | N |
| CCR0085 | Severe | 49 | Male | Hispanic | N/A | N | N | N | N | N | N | N |
| CCR0109 | Severe | 67 | Male | Hispanic | 15.6 | N | Y | N | N | N | N | N |
| CCR0168 | Severe | 75 | Male | Caucasian | 49.6 | Y | Y | Y | N | Y | N | N |
| CCR0180 | Dexa | 65 | Male | Caucasian | 30.7 | Y | Y | Y | Y | N | N | N |
| CCR0191 | Dexa | 35 | Female | Caucasian | 41.2 | N | N | Y | N | N | N | N |
| CCR0195 | Dexa | 70 | Female | African American | 34.4 | Y | Y | Y | N | N | N | N |
| CCR0199 | Dexa | 68 | Female | African American | 51.7 | Y | Y | Y | Y | N | Y | N |

**Table S2.** **Hospitalization course (including information on oxygen device, disease complications, abnormal lab values and relevant medications) of COVID-19 patients included in the study**. Significant increase in ALT and AST are defined as values > 2 x upper normal limit, Leucopenia and lymphopenia defined as values < lower normal limit, Abbreviations: AKI, acute kidney injury; ALT, alanine transaminase; AST, aspartate aminotransferase; NC, nasal cannula; IMV, invasive mechanical ventilation; HFNC, high-flow nasal cannula; N/A, data “not available” in the report. All data were provided by the Center for Health Informatics, University of Cincinnati.

| Patient ID | Cohort | O_2_ device | Complications | | | Abnormal laboratory values | | | | Medications | |
| --- | --- | --- | --- | --- | --- | --- | --- | --- | --- | --- | --- |
|  |  |  | Secondary infections | Shock | AKI | AST↑ | ALT↑ | Leucopenia | Lymphopenia | Remdesivir | Dexamethasone (days given) |
|  |  |  |  |  |  |  |  |  |  |  |  |
| CCR0069 | Mild | None | N | N | N | N | N | N | N/A | N | N |
| CCR0086 | Mild | None | N | N | N | N | N | N | N | N | N |
| CCR0222 | Mild | None | N | N | N | N/A | N/A | N | N | N | N |
| CCR0243 | Mild | None | N | N | N | N/A | N/A | N/A | N/A | N | N |
| CCR0085 | Severe | IMV | Y | N | N | N | N | N | Y | Y | N |
| CCR0109 | Severe | NC | Y | N | N | N | Y | N | Y | N | N |
| CCR0168 | Severe | NC | N | N | N/A | N/A | N/A | N/A | N/A | N | N |
| CCR0180 | Dexa | IMV | Y | Y | Y | N | N | N | N | N | Y (6 days) |
| CCR0191 | Dexa | HFNC | N | Y | N | N | N | N | Y | Y | Y (5 days) |
| CCR0195 | Dexa | NC | N | N | N | N | N | N | Y | Y | Y (6 days) |
| CCR0199 | Dexa | HFNC | N | N | N | N | N | N | N | Y | Y (6 days) |

**Table S3:**  **Viability of cryopreserved COVID-19 patient PBMCs after thawing**. Viability done by flow cytometry using Zombie UV live- dead stain.

| **CCR#** | **Patient cohort** | **%Live** | **Average (%)** | **SD** |
| --- | --- | --- | --- | --- |
| 69 | Mild | 91.8 | **90.3** | 3.2 |
| 86 | Mild | 87.3 |  |  |
| 222 | Mild | 94.1 |  |  |
| 243 | Mild | 88.1 |  |  |
|  |  |  |  |  |
| 85 | Severe | 92.5 | **93.0** | **0.8** |
| 109 | Severe | 92.6 |  |  |
| 168 | Severe | 94.0 |  |  |
|  |  |  |  |  |
| 180 | Dexa | 90.3 | **90.4** | **4.5** |
| 191 | Dexa | 93.9 |  |  |
| 195 | Dexa | 84.1 |  |  |
| 199 | Dexa | 93.3 |  |  |
|  |  |  |  |  |
| D287 | Healthy Control | 98.6 | **97.2** | **1.6** |
| D341 | Healthy Control | 94.6 |  |  |
| D325 | Healthy Control | 97.2 |  |  |
| D354 | Healthy Control | 98.2 |  |  |
| D300 | Healthy Control | 97.5 |  |  |

**Table S4: Immune cell phenotyping flow cytometry panel**

| **Marker** | **Fluorophore** | **Clone** | **Vendor** | **Catalog #** | **RRID** |
| --- | --- | --- | --- | --- | --- |
| Kv1.3 | N/A | N/A | Alomone | APC-101-GP | AB_2340958 |
| IgG | Alexa-Fluor 555 | N/A | ThermoFisher | A-21435 | AB_2535856 |
| Orai1 | ATTO 633 | 3F11/D10/B9 | Alomone | ALM-025-FR | AB_2340986 |
| CD19 | APC-CY7 | HIB19 | Biolegend | 302218 | AB_314248 |
| CD3 | BV 421 | UCHT1 | Biolegend | 300434 | AB_10962690 |
| CD4 | BV 605 | OKT4 | Biolegend | 317438 | AB_11218995 |
| CD8 | BV 711 | SK1 | Biolegend | 344734 | AB_2565243 |
| CD56 | PE Dazzle 594 | HCD56 | Biolegend | 318348 | AB_2563564 |
| CD16 | Alexa Fluor 488 | 3G8 | Biolegend | 302019 | AB_492974 |
| CD14 | BV 510 | 63D3 | Biolegend | 367124 | AB_2716229 |
| Zombie UV | UV | N/A | Biolegend | 423107 | N/A |

**Table S5: List of genes differentially expressed by dexamethasone in the altered calcium signaling pathways (shown in Figure 3C-D).** Differentially expressed genes were determined by comparing the normalized counts for all genes in NanoString Host Response Panel codeset between the severe COVID-19 (n=3) and severe COVID-19 treated with dexamethasone (n=4) patient groups. Comparisons were performed using unpaired t-tests. We identified the differentially expressed genes in the calcium ion signaling-related pathways shown as correlation network in Figure 3C. These genes are presented in this table, along with their annotations and p-values.

|  | **gene** | **Full name** | **Calcium ion signaling pathway** | **p-value** |
| --- | --- | --- | --- | --- |
| 1 | *CXCR4* | C-X-C motif chemokine receptor 4 | Calcium ion mediated signaling  Calcium ion transport | 0.0102 |
| 2 | *CCR5* | C-C motif chemokine receptor 5 | Calcium ion mediated signaling  Calcium ion transport  Calcium ion transmembrane transport  Calcium ion transmembrane import into cytosol  Calcium ion homeostasis | 0.0257 |
| 3. | *FASLG* | Fas ligand (TNF superfamily, member 6) | Calcium ion transport  Calcium ion transmembrane transport  Calcium ion homeostasis  Calcium ion transmembrane import into cytosol | 0.0383 |
| 4 | *VCAM1* | vascular cell adhesion molecule 1 | Calcium ion mediated signaling | 0.0029 |

**Table S6. Values of peak Kv1.3 currents and cell capacitance in untreated or dexamethasone-treated resting HD CD8+T cells.** Data are means ± SD. Statistical significance determined by paired Student’s t-test.

|  | Time | Treatment | |  |  |
| --- | --- | --- | --- | --- | --- |
| Kv1.3 peak current (pA) |  | Vehicle | 1 μM Dexa | P-value | n |
|  | 24 h | 453.93 ± 201.75 | 325.30 ± 218.30 | 0.0076 | 20 cells, 4 donors |
|  | 48 h | 383.23 ± 69.79 | 224.31 ± 62.08 | <0.001 | 18 cells, 4 donors |
|  |  |  |  |  |  |
| Cell capacitance (pF) |  | Vehicle | 1 μM Dexa | P-value | n |
|  | 24 h | 1.04 ± 0.55 | 1.10 ± 0.47 | 0.844 | 20 cells, 4 donors |
|  | 48 h | 0.57 ± 0.46 | 0.76 ± 0.36 | 0.115 | 18 cells, 4 donors |
